# Ethnopharmacological study of traditional medicinal plants used by the people in Metema district, northwestern Ethiopia

**DOI:** 10.3389/fphar.2025.1535822

**Published:** 2025-03-10

**Authors:** Daniel Tadesse, Ermias Lulekal, Getinet Masresha

**Affiliations:** ^1^ Department of Plant Sciences, University of Godar, Gondar, Ethiopia; ^2^ Department of Biology, University of Godar, Gondar, Ethiopia; ^3^ Department of Plant Biology and Biodiversity Management, Addis Ababa University, Addis Ababa, Ethiopia

**Keywords:** ethnobotany, indigenous knowledge, medicinal plants, Metema, ailments

## Abstract

**Background:**

Medicinal plants are used by more than 80% of Ethiopians. The Metema District, shaped by various ethnicities and settlement histories, remains underexplored. This study aimed to document and analyze medicinal plant use and associated traditional knowledge in the local population.

**Methods:**

Data were collected through semi-structured interviews, guided field walks, and focus-group discussions. A total of 110 informants participated in the study, using various sampling techniques. The informant consensus factor (ICF) and direct matrix ranking (DMR) were computed along with descriptive statistics to analyze the basic ethnobotanical data.

**Results:**

In this study, 85 therapeutic plants were utilized to treat 13 disease categories. The three ethnic groups shared 21.18% of their knowledge of medicinal plants. Fabaceae was the most represented family, comprising 11 plant taxa. Herbs were the predominant plant form (42.4%), leaves being the most frequently used (30.5%). Oral administration was the primary method used for the plant extracts (52.3%). Circulatory and blood-related disorders had the highest ICF value (0.91). *Ziziphus spina-christi*, *Ximenia americana*, and *Ficus sycomorus* were ranked as the top multipurpose plants. Fuelwood collection and agricultural expansion have been identified as the major threats to these plants.

**Conclusion:**

This study revealed the rich diversity of medicinal plants and traditional knowledge in the Metema District. The therapeutic potential of the documented plants supports further pharmacological investigations, underscoring the importance of preserving indigenous knowledge and protecting plant resources against ongoing threats.

## Background

Medicinal plants have been central to the treatment of various ailments since ancient times and are a vital component of traditional healing systems. Approximately 70%–80% of the global population still depends on these systems for primary healthcare, driven by their proven effectiveness, cultural relevance, and limited access to modern healthcare (Caniago and Siebert, 1998; Kuniyal et al., 2015).

Globally, the transmission of traditional knowledge to subsequent generations is limited, suggesting the need for measures to safeguard existing traditional wisdom and facilitate its dissemination to future generations (Güler et al., 2021; Karaköse, 2022). In Ethiopia, the use of traditional medicinal plants has a well-established history (Yimam et al., 2022), with much of this knowledge being transmitted orally across generations by healers, knowledgeable elders, and community members (Chekole, 2017). It is estimated that over 80% of Ethiopians rely on traditional medicine, with nearly 95% of these remedies originating from plants (Muluye and Ayicheh, 2020).

Plants are served as major natural resources for traditional as well as modern medicinal systems all over the world (Shakya, 2016). Medicinal plants have been integral to human healthcare for centuries, serving as the primary source of therapeutic agents before the advent of modern pharmaceuticals (Mamun and Khan, 2018). With increasing interest in natural remedies, the study of these plants has gained renewed attention, especially in the context of traditional medicine systems and their pharmacological properties (Li and Weng, 2017). The ethnobotanical study of traditional medicinal plants in the Metema district of northwestern Ethiopia is essential for understanding the rich tapestry of local health practices and the reliance on natural resources for medicinal purposes. In this region, traditional medicinal systems are deeply intertwined with cultural practices, reflecting a profound knowledge of local flora.

Despite the crucial role of traditional medicine and medicinal plants in primary healthcare, efforts to document and promote this knowledge in Ethiopia have been limited (Demie et al., 2018). Only a small portion of the country’s diverse culture and language has been studied thoroughly, to mention a few; Tadesse et al. (2024), Teka et al. (2020), Tugume et al. (2016), Yimam et al. (2022), and Zemede et al. (2024). There is a need for additional surveys across different regions to capture a broader range of sociocultural groups and to preserve unique knowledge and cultural practices (Giday et al., 2007). Research conducted in various Ethiopian regions (Tefera and Kim, 2019; Tuasha et al., 2018) has shown that medicinal plants are increasingly threatened, and the knowledge possessed by the elderly is at risk of disappearing due to inadequate attention.

Due to the ethnopharmacological study of medicinal plants in the world, newly recorded species and different uses of plants may be reported which will contribute to fields such as phototherapy, chemist’s shop, and chemistry (Akbulut et al., 2019; Akbulut et al., 2022; Şen et al., 2022). Thus, the present study will play a role in this regard.

We propose that the sustainable management and conservation of medicinal plants can be achieved when information about their use for treating ailments and traditional herbal practices in specific areas is accessible. It is crucial to preserve this information to benefit both the present and future generations. This study focuses on the Metema district of Northwestern Ethiopia, where we have documented the diversity of ethnomedicinal plants and traditional healing practices among local communities. We anticipate that the qualitative and quantitative insights from this research will support both the conservation and sustainable use of medicinal plants and strengthen the traditional healthcare system. Given Metema’s cultural and ecological landscape and the pressing need to protect its traditional plant knowledge, this study aimed to (i) document the medicinal plants and associated indigenous knowledge utilized by the communities, (ii) assess the major threats facing these medicinal plants, and (iii) identify and report any new ethnomedicinal plant species and practices previously unrecorded.

## Materials and methods

### Description of the study area

**FIGURE 1:**
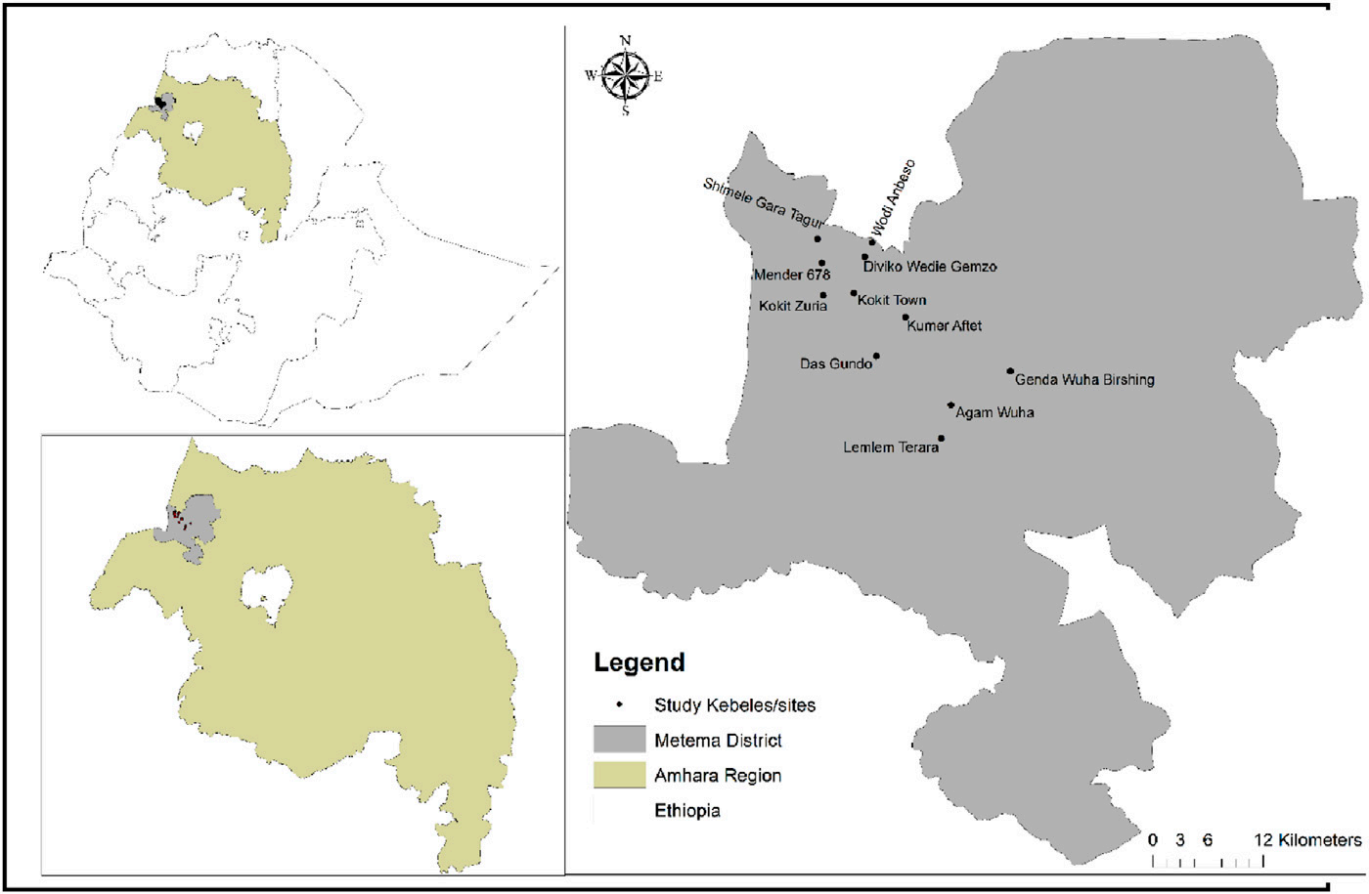
Map of Ethiopia showing the Amhara region and the study site.

This study was conducted in the Metema district of the West Gondar Zone within the Amhara Regional State of Northwestern Ethiopia (Figure 1). Located approximately 925 km northwest of Addis Ababa, Metema comprises 31 kebeles, the smallest administrative units in Ethiopia. Genda Wuha serves as the district’s administrative center. The population of the district is 110,231, consisting of the Agew, Amhara, Gumuz, and Kimant ethnic groups (CSA, 2007). The elevation of the area ranges from 550 to 1,608 m above sea level, covering approximately 440,000 ha (Adamu et al., 2012). Metema is characterized by lowland agroecology, with a mean annual rainfall of 1,008 mm and a monomodal rainfall pattern occurring from June to September (Masresha et al., 2023). The mean annual temperature is 26.2°C, ranging from 15.7°C to 41.0°C. The major crops cultivated in the area include sesame, cotton, and sorghum, while goats and cattle are the primary livestock (Masresha et al., 2023).

According to the broad classification of Ethiopia’s forests, the lowland dry forests in the Metema district fall under the *Combretum-Terminalia* woodland community. The dominant vegetation in this region consists of mixed dry deciduous woodlands, primarily characterized by abundant *Combretum* spp. and *Terminalia* spp. *Communities* (Friis et al., 2010). Other notable species in these woodlands include *Sterculia setigera* Delile, *Boswellia papyrifera* (Caill.) Hochst., *Terminalia leiocarpa* (DC.) Baill., *Lannea fruticose* Engl., *Stereospermum kunthianum* Cham., *Dichrostachys cinerea* (L.) Wight & Arn. and *Pterocarpus lucens* Lepr. Ex Guill. and Perr. (Adamu et al., 2012; Eshete et al., 2011).

### Research design

#### Reconnaissance survey and site selection

The researchers obtained a formal letter from the Vice President for Research and Technology Transfer at the University of Gondar to conduct this study (clearance number 1059/2022). This letter was then presented to the Metema District Administration Office, where we received permission to proceed with a reconnaissance survey and select sample kebeles for the actual study. The reconnaissance survey was conducted from 2nd to 9th July 2022, in Metema district.

Following the reconnaissance survey, 11 kebeles were chosen through stratified random sampling guided by the recommendations of district administrators, local authorities, and elders. These selected kebeles represented 35.48% of the total 31 kebeles in Metema district. The criteria for selecting sample kebeles included the presence of traditional healers, ethnic distribution (Amhara, Agew, and Gumuz), and settlement history (local inhabitants and settlers).

#### Sample size determination and informant selection

Cochran’s formula, as cited by Bartlett et al. (2001), was employed to determine representative samples for the study area. A total of 110 informants were selected (72 males and 38 females), as shown in Table 1. Among these, 27 were key informants and 83 were general informants. The key informants were purposefully selected in consultation with local authorities and elders using the snowball sampling method, whereas general informants were chosen through random sampling, taking into account factors such as age, sex, cultural background, and settlement history.

**TABLE 1:** Description of study kebeles of data collection, highlighting key geographical and demographical attribute.

Study kebele	Gps coordinates		Elevation (m)	Ecology	NI	Gender		Informant type		Ethnicity		
	Latitude	Longitude				M	F	GI	KI	A	G	Ag
Mender 678	12°57′33″N	36°14′58″E	705	Lowland	6	4	2	3	3	0	0	6
Kokit Zuria	12°51′53″N	36°14′42″E	794	Lowland	11	7	4	9	2	11	0	0
Kokit town	12°52′09″N	36°16′00″E	726	Lowland	14	9	5	10	4	14	0	0
Das Gundo	12°44′19″N	36°11′48″E	838	Lowland	13	8	5	11	2	13	0	0
Kumer After	12°48′14″N	36°21′32″E	742	Lowland	7	6	1	3	4	0	7	0
Agam Wuha	12°43′16″N	36°19′15″E	848	Lowland	8	5	3	6	2	8	0	0
Lemlem Terara	12°40′09″N	36°17′40″E	889	Lowland	4	4	0	3	1	4	0	0
Diviko Wedie Gemzo	12°57′48″N	36°19′17″E	764	Lowland	29	17	12	24	5	29	0	0
Shimele Gara Tagur	13°01′12″N	36°17′00″E	740	Lowland	10	7	3	8	2	10	0	0
Genda Wuha Birshign	12°44′46″N	36°26′38″E	764	Lowland	4	2	2	3	1	4	0	0
Wedi Anbesso	12°58′29″N	36°24′40″E	849	Lowland	4	3	1	3	1	4	0	0
Total					110	72	38	83	27	97	7	6

NI, number of interviewees, Gender (M = male, F = Female), Informant type (GI, general informant; KI, Key Informant), Ethnicity (A = amhara, G = gumuz, Ag = Agew).

### Data collection

Ethnobotanical data were gathered through face-to-face interviews with informants. To ensure consistency, pre-planned semi-structured questionnaires and standardized data collection protocols (Alexiades, 1996; Martin, 2004; Heinrich et al., 2009) were employed. Field trips were conducted between August 2022 and October 2023 to identify and gather therapeutic plant species used within the district. Most interviews were conducted in Amharic, the region’s common language; however, local translators assisted when the informants spoke other languages. All collected information was later translated into English. The questionnaire focused on the local names, plant types, parts used, ailments treated, and preparation and administration methods.

For additional on-site data collection, guided field walks were conducted with informants. During these walks, plant specimens were collected, and details of plant names, plant habits, habitats, conservation status, and other relevant attributes were documented. Four focus group discussions were held with seven key informants in each session, addressing topics such as threats to medicinal plants, conservation practices, antidotes, and dosage, following Martin’s (1995) approach. These discussions helped validate the data gathered from the individual informants.

### Specimen identification

The voucher specimens collected were authenticated by consulting taxonomic literature, reference voucher specimens, and various Flora of Ethiopia and Eritrea books. Specimen identification was performed at the National Herbarium (ETH) of Ethiopia at Addis Ababa University and the Herbarium of the University of Gondar under the guidance of an expert. The names of the species, genera, and families were further validated using the Plants of the World Online website (https://powo.science.kew.org). Finally, the identified specimens were dried, pressed, mounted on herbarium sheets, and deposited at the Herbarium of the Department of Biology, University of Gondar, Gondar, Ethiopia.

### Data analysis

This study used a combination of qualitative and quantitative ethnobotanical methods (Martin, 1995; Cotton, 1996). To compare knowledge of medicinal plants across social groups in the Metema district, data analysis was conducted using SPSS (version 29). A t-test was conducted to explore the differences in medicinal plant knowledge based on settlement history (locals vs settlers), gender (men vs women), education level (literate vs illiterate), and healing experience (key informants vs general informants). Additionally, one-way ANOVA was applied to assess significant differences in the mean knowledge of medicinal plants across various age groups and ethnic backgrounds. Medicinal plant knowledge was assessed in terms of the number of medicinal plants mentioned by each informant. Microsoft Excel (2013) was used for calculating totals and percentages, and for creating tables and graphs.

#### Informant consensus factor (ICF)

The Informant Consensus Factor (ICF) measures the consensus or homogeneity in ethnobotanical knowledge shared by informants (Heinrich et al., 1998). This was calculated using the following formula:

$$\text{ICF}=\frac{\text{Nur}\;–\;\text{Nt}}{\text{Nur}-1}$$

Where Nur is the total number of use reports for each disease category and Nt is the number of species used within that category. The ICF value ranges from 0 to 1, where 0 indicates no shared knowledge or exchange of use information among informants and 1 reflects a high level of consensus or knowledge exchange.

#### Direct matrix ranking (DMR)

The Direct Matrix Ranking (DMR) method was used to compare the multipurpose medicinal plants frequently mentioned by informants. This approach helps score the diversity of uses for selected medicinal plants (Martin, 1995; Cotton, 1996). Eight multipurpose plant species were chosen based on use citations along with eight common uses identified in the study area. Twelve key informants were selected to independently rate each plant’s utility and assign scores (5 = best, 4 = very good, 3 = good, 2 = less used, 1 = least used, 0 = not used). The scores from each participant were summed and averaged for each plant and use category. Finally, the aggregate values for each species and use category were calculated and ranked.

## Results

### Socio-demographics features of the informants

Demographic characteristics were documented based on the information provided by the participants. A total of 110 informants were interviewed: 72 men (65.45%) and 38 women (34.55%). A significant difference (p < 0.05) was found between key and general informants regarding the mean number of medicinal plants known and used in the Metema district; key informants demonstrated greater knowledge, averaging 13.6 plants, compared to general informants, who averaged 5.8 plants. Similarly, there was a notable difference (p < 0.05) in the mean knowledge of medicinal plants between settlement histories (locals and settlers) and sex (men and women). However, no significant difference (p > 0.05) was observed between literate and illiterate respondents. One-way analysis of variance (ANOVA) indicated a significant difference (p < 0.05) in the mean number of medicinal plant species reported among different age groups. Additionally, significant differences (p < 0.05) were observed in the average number of medicinal plants reported among different ethnic backgrounds, with the Gumuz community mentioning more medicinal plants on average (14.6) than the Agew (10.2) and Amhara (7.1) groups (Table 2).

**TABLE 2:** Medicinal plant knowledge of the informants in Metema district (n = 110).

Parameter	Informant categories	Number of informants	Mean ± SD	Test statistic	*p* value
Healing experience	Key Informants	27	13.6 ± 3.0	145.798	0.000*
	General Informants	83	5.8 ± 2.9		
Settlement history	Local	40	8.6 ± 4.7	6.887	0.010*
	Settlers	70	6.3 ± 3.7		
Gender	Male	72	8.6 ± 4.7	8.018	0.006*
	Female	38	6.1 ± 3.6		
Education level	Illiterate	42	7.6 ± 4.7	0.121	0.729
	Literate	68	7.9 ± 4.3		
Age	20–39	46	5.1 ± 2.1c	19.800	0.000*
	40–59	35	7.6 ± 4.4 b		
	≥60	29	11.2 ± 4.4a		
Ethnic background	Amhara	97	7.1 ± 3.9 b	12.234	0.000*
	Gumuz	7	14.6 ± 5.4a		
	Agew	6	10.2 ± 4.8 b		

*Shows a significant difference at p < 0.05.

### Medicinal plant diversity

**FIGURE 2:**
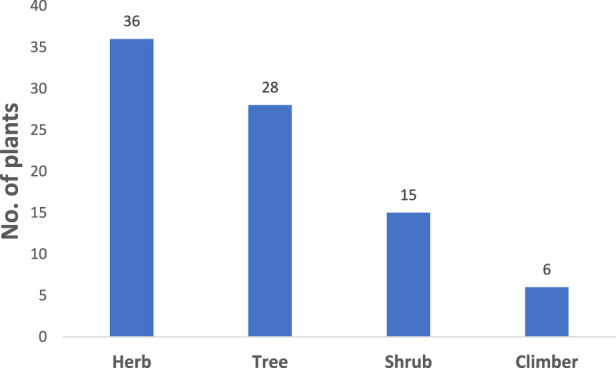
Distribution of medicinal plants in different life forms.

A total of 85 medicinal plant taxa belonging to 71 genera and 33 families were identified and documented for their use in traditional human ethnomedicine across the three ethnic groups studied (Supplementary Table S1). Among these families, 17 (51.5%) included two or more species, whereas the remaining 16 families (48.5%) were represented by a single species. The Fabaceae family had the highest number of species (11 species), followed by Malvaceae (8 species), and Cucurbitaceae (7 species). Life form analysis revealed that herbs constituted the largest group, with 36 species identified, followed by trees (28 species), shrubs (15 species), and climbers (6 species) (Figure 2).

### Plant parts and conditions for preparing remedies

**FIGURE 3:**
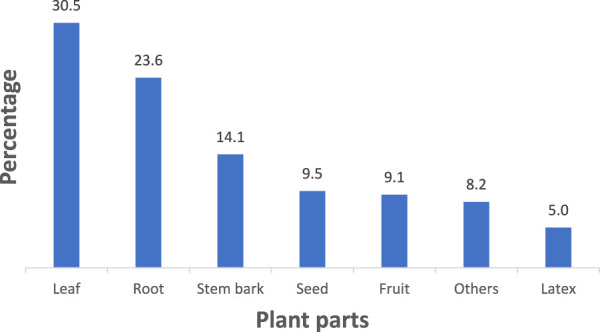
Plant part used in the preparation of herbal medicine.

Various plant parts were reported to be utilized in remedy preparation within the district (Figure 3). The most commonly used plant part was the leaves, which accounted for 30.5% of the total, followed by the roots at 23.6%. Other plant parts, including bulbs, tubers, root bark, twigs, seed oil, flowers, gum, and stems collectively constituted 8.2%. Most remedies were prepared using freshly collected plant parts (64.1%), while 22.3% were made using either fresh or dry parts, and 13.6% were prepared exclusively from dry parts.

### Ailments treated with medicinal plants

The study identified 67 human ailments treated with 85 medicinal plant species found in the district (Supplementary Table S1). Among these species, the majority (62 species, 72.1%) were reported to address two or more ailments, whereas 24 species (27.9%) were used for single ailments. Notable versatile plants include *Withania somnifera*, which treats nine different ailments; *Moringa stenopetala,* which addresses eight ailments; and *Calotropis procera*, which is effective for seven ailments. Additionally, *Ziziphus spina-christi*, *Securidaca longepedunculata,* and *Azadirachta indica* have been reported to treat six ailments.

In addition to individual plant species, traditional healers in the study area frequently combine multiple parts to create remedies for various ailments. A total of 16 plant species were used in two or more combinations for this purpose. For example, the roots of *Carissa spinarum* are crushed with the roots of *Ruta chalepensis* and the bulb of *Allium sativum*, and water is added to the mixture and drunk to treat snakebite. To address febrile illness, the leaves of *Ficus sycomorus*, *Cordia africana,* and *Zehneria scabra are* mixed, and boiled, and the filtrate is consumed and used for fumigation. Furthermore, the roots of *S. longepedunculata*, *Tacazzea venosa*, *W. somnifera*, *Sida rhombifolia,* and *Clerodendrum alatum* are pounded, heated, and inhaled to treat evil eyes. The root of *Pterolobium stellatum* is crushed and mixed with the bulbs of *A. sativum* and *Zingiber officinale*, along with the root of *R. chalepensis*, and sniffed as a remedy for the evil eye.

### Pharmacological values of selected medicinal plants

The pharmacological values of medicinal plants have garnered increasing attention in traditional medicine, particularly for their diverse therapeutic applications. Among the 85 therapeutic plants reported in this study, the four top-ranked medicinal species included *Z. spina-christi*, *Ximenia americana*, *F. sycomorus*, and *T. leiocarpa*, which had shown important medicinal properties that could be used to treat a wide range of health problems.

*Ziziphus spina-christi* demonstrates significant pharmacological potential and was widely utilized in traditional medicine for various ailments. It was particularly effective in treating uvulitis; the stem bark was crushed, mixed with cold water, and consumed. For stomachaches, the roots were prepared in a similar manner, providing relief when the fluid was ingested. The leaves were also employed against ringworm, applied directly to the affected area using unripe fruit. For dandruff, the leaves were pounded, the fluid was sieved, diluted with water, and then applied to the scalp. Additionally, the roots can be used to combat malaria by crushing them, mixing them with water, and drinking the solution. Lastly, the leaves served as a remedy for spider poison, where the extracted fluid was painted on the affected area.

*Ximenia americana* exhibited notable pharmacological properties in treating a variety of conditions. For bleeding wounds, both fresh and dry stem bark can be crushed into a paste for topical application. Eye infections were addressed using the same part of the plant; the bark was crushed and homogenized with warm water to create a fumigating solution. In cases of uvulitis, the fresh stem bark was crushed, mixed with water, and consumed to provide relief. Additionally, fresh leaves were used for treating uvulitis in infants by placing them on the baby’s head while reciting the phrase “Return it, return it back” repeatedly, showcasing the plant’s cultural significance in traditional healing practices.

*Ficus sycomorus* presents a range of medicinal applications effective in treating various ailments. For febrile illnesses, fresh leaves were boiled with Cordia africana and *Z. scabra*, and the resultant filtrate was both consumed and used to wash the entire body. In instances of sudden sickness, fresh leaves were boiled with *Z. scabra*, and the mixture was employed for a full-body wash. The fresh latex from the plant served as a topical treatment for wound healing and was also effective against spider poison when applied to the affected area. Furthermore, the fresh stem bark was used for snakebite treatment by crushing it and forming a paste for application.

*Terminalia leiocarpa* offered a variety of medicinal applications, particularly in traditional healthcare practices. For the treatment of uvulitis, pieces of fresh or dry stem bark were placed on the patient’s head while reciting the phrase “put up, put up.” To address tapeworms, the fresh stem bark was crushed, mixed with water, and ingested. For dysuria, the fresh stem bark was crushed, boiled with water, and sweetened with sugar before consumption. In cases of jaundice, the crushed fresh stem bark was combined with boiling *Cicer arietinum*, and the filtrate was consumed. Lastly, for amoebiasis, the fresh stem bark was crushed, boiled, and the decoction was ingested once cooled.

### Methods of preparation and route of administration of remedies

**FIGURE 4:**
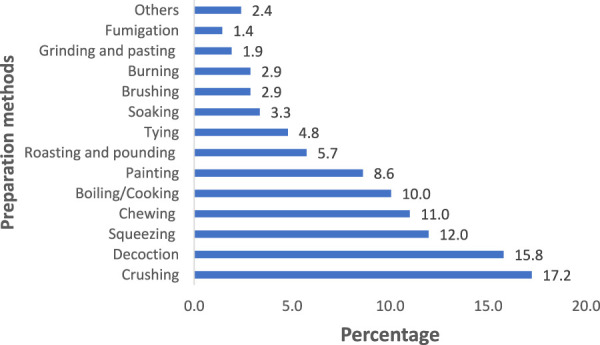
Methods of remedy preparation in Metema district.

The communities in the study area have employed a range of methods to prepare and administer traditional medicinal remedies. The most prevalent preparation methods were crushing (17.2%), decoction (15.8%), squeezing (12%), chewing (11%), and boiling/cooking (10%). Five other preparation methods collectively accounted for 2.4% of the remedies used (Figure 4).

**FIGURE 5:**
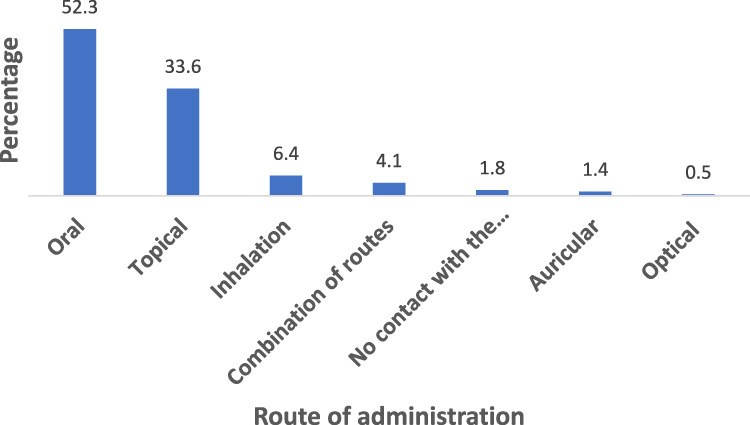
Route of administration of medicinal plants in Metema district.

Regarding the routes of administration, over half of the remedies (115 preparations, or 52.3%) were administered orally (Figure 5). This was followed by topical application, which accounted for 33.6% of the preparations. Traditional healing practices also involve administering remedies without any physical contact with the ailment, accounting for 1.8% of cases. For example, during the circumcision of a young boy, the removed tissue was covered with the stem bark of *C. africana* to promote wound healing. Similarly, to treat uvulitis, the dried fruit of *Lagenaria siceraria* was hung in a house. Furthermore, certain traditional methods are believed to be effective in dispelling evil spirits. For instance, the root of *Pappea capensis* is burned to fumigate the area with the belief that this will prevent evil entities. Likewise, the root of *S. longepedunculata* is also placed in a fire, under the belief that it will prevent snakes from approaching.

### Ethnobotanical knowledge distribution among ethnic groups

**FIGURE 6:**
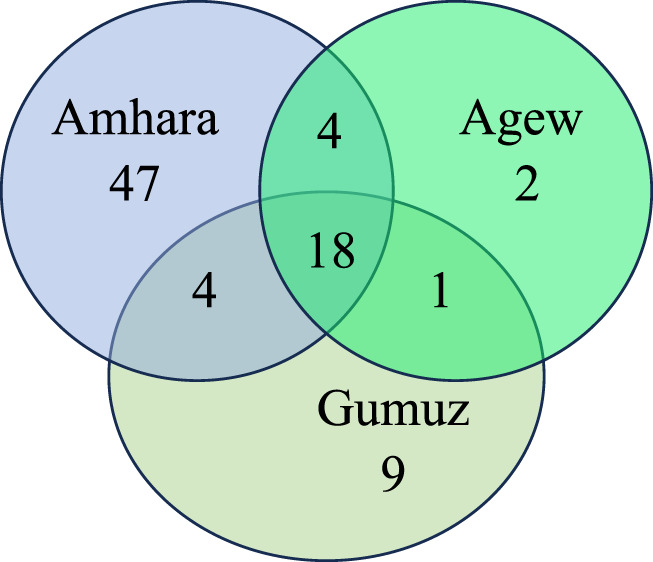
Venn diagram of medicinal plants among the studied ethnic groups in Metema district.

The ethnobotanical knowledge of the three ethnic groups—Amhara, Agew, and Gumuz was compared and illustrated using a Venn diagram (Figure 6). The Agew, Gumuz, and Amhara ethnic groups reported 25, 32, and 73 plant species, respectively. Pairwise comparisons indicated that the Amhara and Gumuz groups, as well as the Amhara and Agew groups, exhibited the highest degree of similarity, sharing 4.70% of their medicinal plant species. In contrast, the Agew and Gumuz groups had the least overlap, sharing only 1.18% of their medicinal plant species. Overall, the three ethnic groups collectively utilized 18 species of medicinal plants (21.18%) across the district. Notably, the Amhara ethnic group uniquely used 55.3% of medicinal products, while the Gumuz and Agew ethnic groups accounted for 10.6% and 2.3% of uniquely used species, respectively.

### Informant consensus factor

ICF was calculated to identify the most effective medicinal plants for treating common ailments in the district. ICF values were determined based on disease categories adapted from the International Classification of Diseases (WHO, 2023). In total, 13 disease categories were identified, with ICF values ranging from 0.53 to 0.91, where the maximum possible value is 1.0 (Table 3). The disease categories with the highest ICF values were those related to the circulatory system and blood/blood-forming organs, with an ICF of 0.91. This was closely followed by diseases and symptoms involving the nervous system with an ICF of 0.89. In contrast, the category of diseases and symptoms related to the respiratory system had the lowest ICF value (0.53).

**TABLE 3:** ICF values of medicinal and magical plants for treating human ailments in Metema district.

Disease categories	Reported diseases (user reports are in bracket)	No. of species	Use citations	ICF
Diseases of the circulatory system and blood and blood-forming organs	Hypertension (29), Anemia (3), heart disease (2)	4	34	0.91
Diseases and symptoms involving the nervous system	Headache (4), snakebite (27), scorpion sting (14), spider poison (22), febrile illness (168), tendinitis (1)	28	236	0.89
Infectious and parasitic diseases	Rabies (4), tapeworm (8), amoebiasis (4), malaria (71), intestinal parasite (20), ring worm (24), scabies (10), uvulitis (23), wart (8), bacterial infection on the tip of a finger (7), dandruff (7), nail fungus (1)	31	187	0.84
Injury, poisoning, and certain other consequences of external causes	Circumcision wound (8), ear pest (2), wound healing (42), bleeding wound (45), dirt on the eye (1), wound on the penis (3)	18	101	0.83
Endocrine, nutritional, and metabolic diseases and Neoplasms	Diabetes mellitus (18), Cancer (7)	5	25	0.83
Diseases and symptoms involving the digestive system	Toothache (1), stomachache (14), constipation (17), sudden sickness (31), abdominal bloating (16), gastritis (11), diarrhea (11), gum bleeding (5), gum disease (16), jaundice (43), rectal prolapse (2), teething in babies (2)	38	169	0.78
Disease of the skin	Hair loss (3), skin disease (2)	2	5	0.75
Symptoms, signs, and clinical findings not elsewhere classified	Snake repellent (1), evil eye (34), evil spirit (3), to eradicate devil (2)	12	40	0.72
Diseases of the musculoskeletal system or connective tissue	Gout (4), rheumatism (4), back pain (15), stabbing pain (4), Osteoporosis (1), tendon issue (1)	10	29	0.68
Pregnancy, childbirth, and the puerperium	Retained placenta (3), miscarriage (3), uterus sore (1)	3	7	0.67
Urogenital conditions	Male erectile dysfunction (4), satisfying sexual desire (2), Dysuria (2), kidney disease (1)	4	9	0.63
Diseases of the ear or mastoid process and visual system	eye infection (2), ear lesion (4)	3	6	0.60
Diseases and symptoms involving the respiratory system	Asthma (3), cough (7), common cold (7), tonsilitis (2), Covid (1)	10	20	0.53

### Direct matrix ranking exercise

The DMR exercise was conducted in the Metema district to evaluate and prioritize the most important medicinal plant species utilized by local communities (Table 4). The findings from the DMR exercise revealed that *Z. spina-christi*, *X. americana*, and *F. sycomorus* were the top-ranked medicinal plant species. Furthermore, the DMR exercise assessed various categories of use of these plants in the district. Accordingly, the most common categories of use included medicinal applications, fencing materials, and livestock forage.

**TABLE 4:** DMR of eight multipurpose medicinal plant species in the Metema district.

Medicinal plants	Use categories								Total	Rank
	Co	Fu	LF	Md	Fo	FW	FI	Fe		
*Azadirachta indica*	3.6	2.2	1.4	4.1	0.9	2.7	2.8	3.4	16.3	8th
*Stereospermum kunthianum*	1.8	1.9	1.9	4.0	0.0	2.0	3.5	2.6	24.6	5th
*Terminalia leiocarpa*	5.0	3.0	3.3	1.7	0.0	4.5	3.5	4.0	28.0	4th
*Ficus sycomorus*	2.8	3.2	4.7	3.4	4.3	3.3	2.1	2.5	29.0	3rd
*Carissa spinarum*	1.8	0.6	1.3	3.3	3.8	2.8	0.2	4.3	19.8	6th
*Ziziphus spina-christi*	3.9	4.3	5.0	1.7	4.7	4.1	3.8	5.0	36.3	1st
*Ximenia americana*	2.2	2.5	2.8	4.1	4.9	3.3	2.5	3.7	30.0	2nd
*Moringa stenopetala*	0.1	0.1	4.4	4.8	4.3	1.8	0.0	0.9	19.5	7th
Total	21.1	17.7	24.8	27.0	22.8	24.3	18.3	26.3		
Rank	6th	8th	3rd	1st	5th	4th	7th	2nd		

N.B., The scores in the table indicate the average values of ranks given to medicinal plants based on their use diversity. Co, Construction; Fu, Furniture; LF, livestock forage; M, md; Fo, Food; FW, fuel wood; Farm implements = FI, Fence = Fe.

### Novel ethnobotanical findings

The current ethnobotanical study in the Metema district has revealed several novel findings. Notably, the research team identified six plants that were being used as traditional medicines for the first time in Ethiopia (Table 5). Among these newly documented medicinal plants, five have been recognized for their medicinal uses in other regions of the world. However, one species, *T. venosa*, has not been linked to any known medicinal application prior to this study.

**TABLE 5:** Plants reported to treat ailments in Metema district for the first time in Ethiopia.

Scientific name	Ailments treated in the current study	Reports from other parts of the world
*Phaseolus lunatus*	Wound healing, Spider poison	Magalhãesa et al. (2019)
*Cucumis melo*	Spider poison, Scorpion sting	Anwar et al. (2024)
*Cucumis metuliferus*	Wound on penis	Mzena et al. (2018)
*Tacazzea venosa*	Evil spirit	-
*Oryza sativa*	Diarrhea	Gedif and Hahn (2003)
*Dioscorea dumetorum*	Diarrhea, Diabetes mellitus	Bukatuka et al. (2016)

### Threats to medicinal plants and conservation efforts

An ethnobotanical study in the Metema district identified several threats to the sustainability of medicinal plant resources and the indigenous knowledge associated with them. During group discussions, participants highlighted seven key threats: the use of herbicides and insecticides, human-induced fires, agricultural land expansion, construction activities, fuelwood collection, the use of plants for agricultural implements, and overgrazing. Additionally, participants noted other threats, including the use of medicinal plants for household utensils, informal cross-border trade to neighboring Sudan, deforestation, seasonal migration patterns, and climate change-related events, such as drought. These discussions also revealed a lack of effective management practices for medicinal plants.

To assess the severity of these threats, we conducted interviews with 12 key informants, who ranked the seven prioritized threats to medicinal plants. The range of values for prioritization was from 23 to 79. The findings indicated that fuelwood collection was the most significant threat, followed by agricultural land expansion and the use of herbicides and insecticides (Table 6).

**TABLE 6:** Ranking of threats to medicinal plants in Metema district.

Threats	Key informants												Total score	Rank
	KI1	KI2	KI3	KI4	KI5	KI6	KI7	KI8	KI9	KI10	KI11	KI12		
Use of herbicides and insecticides	2	2	3	1	6	6	2	3	5	5	6	6	47	3rd
Human-induced fire	3	3	1	3	5	5	4	4	4	4	4	4	44	4th
Agricultural land expansion	7	7	7	7	4	4	6	6	6	7	5	5	71	2nd
Construction	4	5	4	5	1	1	5	5	3	3	3	3	42	5th
Fuel wood	6	6	6	6	7	7	7	7	7	6	7	7	79	1st
Farm implements	1	1	2	2	2	2	3	2	2	2	2	2	23	7th
Over grazing	5	4	5	4	3	3	1	1	1	1	1	1	30	6th

## Discussion

### Socio-demographics of the informants

This ethnobotanical study in the Metema district provided valuable insights into how socio-demographic factors influence the knowledge and use of medicinal plants within the local community. The findings indicate that men possess greater knowledge of medicinal plants than women do, suggesting that men generally have a broader understanding of these resources. This may be due to the men’s increased exposure to social affairs in the study area, which offers more opportunities for knowledge acquisition. This finding aligns with those of Chekole et al. (2015) and Kefalew et al. (2015), who reported similar results. The result may also be attributed to the greater number of males included in the present and cited studies.

Age also plays a significant role in knowledge of medicinal plants. Older informants, particularly those aged 60 years and above, exhibited a higher level of knowledge than younger age groups (20–39 years and 40–59 years). This suggests that firsthand experience and long-term familiarity contribute to the generation’s deeper understanding of medicinal plants, a finding that is consistent with that of Tahir et al. (2021), who emphasized the value of accumulated cultural exposure, experience, and exchange over time. However, studies have noted an alarming trend: as older knowledgeable individuals pass away, their wisdom risks are lost because of limited transmission to the next-generation (Demie et al., 2018; Nigussie and Kim, 2019; Karaköse, 2022; Tahir et al., 2023). This loss is compounded by the younger generation’s declining interest in learning from elders, endangering the preservation of indigenous knowledge.

The educational background appeared to have a limited impact, as both literate and illiterate individuals exhibited comparable levels of knowledge. However, key informants, such as herbal practitioners, identified more medicinal plants than did general informants, highlighting their specialized knowledge and direct experience with these plants, as supported by Tahir et al. (2023).

Interestingly, local inhabitants cited more medicinal plants than settlers, likely because of their longer residency and greater familiarity with the local flora. Among the ethnic groups in the study area, the Gumuz people have emerged as the most knowledgeable about medicinal plants. This expertise is attributed to their strong reliance on natural resources for health and daily survival, as well as the exchange of indigenous knowledge both within their community and with neighboring groups in Sudan.

### Medicinal plant diversity

This study documented a rich diversity of medicinal plants used for treating human ailments in the Metema district, identifying a total of 85 medicinal plant species. Compared to findings from other studies conducted in Ethiopia and internationally, this number was notably higher. For instance, ethnobotanical research by Chekole (2017), Regassa et al. (2017), Tefera and Kim (2019), Tadeyos and Wendawek (2022), and Mekuria and Abduro (2022) recorded 51, 25, 70, 62, and 43 medicinal plant species, respectively, across various regions in Ethiopia. In studies outside Ethiopia, researchers like Ojha et al. (2020), Ishtiaq et al. (2015), Tugume et al. (2016), Wiryono et al. (2019), Al-Robai et al. (2022), and Akbulut et al. (2019), documented 70, 10, 27, 9, 21, and 48 medicinal plant species, respectively, in counties including India, Indonesia, Uganda, Pakistan, Saudi Arabia, and Turkey. The study documented medicinal plants were also found lower than other studied (Karaköse, 2022; Şen et al., 2022).

A study conducted in Quara (Tadesse et al., 2024), which is in close proximity to the current study area, reported 128 medicinal plant species, a number higher than that observed in the present investigation. This discrepancy can be attributed to the fact that Quara district encompasses two agroecological zones, lowland and highland, which contribute to the presence of extensive vegetation cover. In contrast, the current study was limited to lowland areas exclusively.

Among the identified medicinal plants, the Fabaceae family was dominant. This prominence may be attributed to the unique adaptations of Fabaceae species to their local environment, such as their symbiotic relationships, which enable them to thrive in nitrogen-deficient soils. Their extensive root systems also allow these plants to outcompete other plants for resources, giving them a competitive edge in the region (Getaneh et al., 2023; Hedberg et al., 2006). This finding aligns with those of previous ethnobotanical studies conducted in Ethiopia (Kidane et al., 2018; Nigussie and Kim, 2019; Tahir et al., 2023), and other regions of the world (Bhandari et al., 2021; Cordero et al., 2022; Masters, 2023).

In terms of plant habits, herbs were found to be the primary type utilized by the people of the Metema district, likely due to their wide availability, market accessibility, and perceived therapeutic effectiveness. This trend is consistent with the findings reported in other studies (Agize et al., 2022; Faruque et al., 2019; Ojha et al., 2020; Tamene et al., 2024).

### Plant parts and the conditions for preparing remedies

The study found that leaves were the most commonly used plant parts for remedy preparation, followed by the roots. This pattern aligns with the findings of other studies in Ethiopia (Ashagre and Lulekal, 2021; Demie et al., 2018; Nigussie and Kim, 2019; Tahir et al., 2021) and globally (Amjad et al., 2020; Cordero et al., 2022; Akbulut et al., 2019; Karaköse, 2022; Şen et al., 2022). Leaves are often preferred owing to their accessibility, abundant availability, and high concentrations of bioactive compounds. Moreover, harvesting leaves has a minimal impact on the survival of plant species, which helps to maintain ecological sustainability. Although roots have been reported as the most frequently used plant part in some studies (Bhandari et al., 2021; Chekole et al., 2015; Teklehaymanot and Giday, 2010) and the second most used plant part in the current study, heavy reliance on roots poses risks to the long-term survival of these medicinal plants (Balde et al., 2015).

Consistent with previous Ethiopian studies (Abera, 2014; Lulekal et al., 2013; Tahir et al., 2023), most remedies in the Metema district were prepared using fresh plant parts. The preference for fresh materials is generally linked to the enhanced efficacy of their bioactive compounds (Chekole et al., 2015). However, given that many medicinal plants in the study area are herbs, remedy availability may be seasonal. Herbs in this dryland region typically grow for only a few months, limiting access to fresh materials outside the growing season.

### Ailments treated with medicinal plants

Analysis of medicinal plant diversity in the Metema district showed that nearly three-quarters of the identified species were used to treat multiple diseases. This widespread application suggests a high level of adoption and reliance on these plants within the local community, consistent with the findings of Pala et al. (2019). The versatility of these medicinal plants in treating various health conditions may be due to the combined action of multiple bioactive constituents. Supporting this idea, Mahomoodally (2013) noted that the synergistic effects of diverse medicinal components can enhance the catalytic activity and improve the absorption of beneficial compounds in the human body. These findings highlight the importance of further scientific investigation of the phytochemical profiles and pharmacological properties of these versatile plants. Understanding the mechanisms that enable certain medicinal plants to treat a broad range of ailments could significantly advance traditional medicine practices and offer more effective and holistic treatment options. Such insights have the potential to improve the overall health and wellbeing of the local population by preserving and enhancing indigenous health practices.

### Pharmacological values of some selected plants

The study’s four highest-ranked medicinal species have been documented as therapeutically effective in other studies as well. *Ziziphus spina-christi* leaves have been shown to be effective against dandruff (Yimam et al., 2022). *Ximenia americana* fruit has been found to alleviate fever, toothache, and common cold symptoms (Zemede et al., 2024). *Ficus sycomorus* leaves have demonstrated efficacy in treating stomachache (Zemede et al., 2024). The root of *T. leiocarpa* has been utilized to address scorpion stings and malaria, while its stem bark has been employed in the treatment of jaundice, scorpion stings, and uvulitis (Tadesse et al., 2024).

A review of the eight multipurpose medicinal plants selected for the direct matrix ranking exercise was conducted, focusing on their *in vivo* experiments as documented in the literature (Table 7). The findings indicate that *in vivo* experiments have been performed for all selected species, aligning with the traditional medicinal uses reported in the current study. This consistency underscores the compatibility between the scientific research and the traditional knowledge identified.

**TABLE 7:** Review of an *in vivo* experiment with selected medicinal plants.

Medicinal plants identified	Review of an *in vivo* experiment
*Azadirachta indica*	Anti-plasmodial activity of both aqueous and ethanolic leaf extracts were studied *in vivo* using *Plasmodium berghei* infected mice and demonstrated significant anti-plasmodial activity (Oseni and Akwetey, 2012). This is in agreement with our report of the use of this species for treatment of malaria
*Stereospermum kunthianum*	Maceration the stem bark in 80% v/v methanol extract was analyzed for its acute toxicity profile in rats for its effects on wound healing and found significant reduction of wound area (Nwinyi et al., 2021). This is in agreement with our report of the use of this species for treatment of wound healing
*Terminalia leiocarpa*	The root bark extract therapeutic activity was carried out against *E. coli* in experimentally infected rats, and found a significant reduction of defecation frequency, water content of faeces, weight and volume of diarrheic faeces, and *E. coli* load of faeces (Adrien et al., 2018). This is in agreement with our report of the use of this species for treatment of amoebiasis whose major symptom is diarrhea
*Ficus sycomorus*	An *in vivo* toxicological analysis of the fruit of the plant on Wistar rats showed positive results as enzymatic examination of the liver, kidney and blood of the Wistar rats did not indicate any serious damage (Braide et al., 2018). Additionally, an *in vitro* analysis of the fruit extract showed bactericidal effect on multi drug resistant bacteria. This is in agreement with our report of the use of this species for treatment of wound healing by preventing bacterial growth
*Carissa spinarum*	An *in vivo* methanolic extract of root was evaluated on burn wound model in mice, showed significant wound healing activity. Besides, an *in vitro* antimicrobial activity of the methanolic root extract against the bacterial and fungal strain using agar dilution method also exhibited significant antimicrobial activity against all the tested microorganisms (Sanwal and Chaudhary, 2011). This is in agreement with our report of the use of this species for treatment of snakebite, as it helps to heal the wound caused by the bite
*Ziziphus spina-christi*	The root bark was evaluated for *in vivo* anti plasmodial activity against Plasmodium berghei in mice. It showed a potent activity against the parasite suggesting the plant as promising agent for malaria treatment (Adzu et al., 2007). This agrees with our report of the use of this species for treatment of malaria
*Ximenia americana*	An *in vivo* extract of stem bark induces a decrease in mast cell concentration leading to the healing process of skin wound contraction in rats (Castro Souza et al., 2017). This agrees with our report of the use of this species for treatment of bleeding wound
*Moringa stenopetala*	Crude aqueous leaf extract of the plant in an *in vivo* experiment carried out on male guinea Pigs had shown blood pressure lowering effect substantiating the use of the plant in traditional medicine (Mengistu et al., 2012). This agrees with our report of the use of this species for treatment of hypertension

### Methods of preparation and route of administration of remedies

This study found that the most common methods of preparing medicinal remedies in the Metema district included crushing, decoction, squeezing, and chewing. These preparation methods align with the findings of previous ethnobotanical studies in various regions (Alemu et al., 2024; Amsalu et al., 2018; Atnafu et al., 2018). Informants reported that no standardized approach exists for administering remedies; instead, the method varies based on factors such as patient age and health condition. This observation is consistent with Ahmad et al. (2014), who noted the flexible, patient-specific nature of traditional medicine practices.

Oral administration was identified as the primary route for delivering herbal remedies in the Metema district, a trend observed in other ethnobotanical studies that also reported that the oral route was predominant in traditional medicines (Regassa, 2013; Tahir et al., 2023). The range of preparation techniques and administration routes highlights the depth of traditional knowledge within the local community. Healers are likely to adapt their methods based on the specific needs and conditions of each patient, drawing on a rich body of experiential knowledge accumulated over generations.

### Ethnobotanical knowledge distribution among ethnic groups

This study revealed that 21.18% of medicinal plants were mentioned across all three ethnic groups. However, the Amhara ethnic group reported a higher number of unique medicinal plant species than the Agew and Gumuz groups. This may be due to the large population size and greater agricultural land coverage in the area. Additionally, the seasonal movement of the Amhara people from neighboring highland districts to Metema for farming and livestock rearing may have fostered knowledge sharing of medicinal plants within their community. In contrast, the Agew ethnic group reported fewer medicinal plants, possibly because of the secretive nature of their traditional knowledge, a characteristic commonly observed in many indigenous communities. These findings, along with those of previous studies (Haq et al., 2022; Sop et al., 2012; Teka et al., 2020), highlighting that the diversity of medicinal plant knowledge is often shared by ethnicity and cultural backgrounds. These insights emphasize the importance of considering ethnic and cultural contexts when documenting and interpreting traditional medicinal plant knowledge.

### Informant consensus factor

This study reported ICF values ranging from 0.53 to 0.91, indicating a strong consensus among the informants regarding the therapeutic uses and effectiveness of specific medicinal plant species, which is consistent with the findings of Tuttolomondo et al. (2014). The highest ICF value (0.91) was observed under conditions related to the circulatory system and blood/blood-forming organs. This high level of agreement aligns with the findings of previous studies (Cheng et al., 2022; Ralte et al., 2024; Zemede et al., 2024) and suggests that the local community holds strong confidence in the efficacy of certain plants for treating these conditions, specifically hypertension, anemia, and heart disease. The second-highest ICF value was recorded for ailments related to the nervous system, which is consistent with the findings of Zemede et al. (2024), who reported a high consensus for conditions such as headaches and febrile illnesses in this category. The third highest ICF value was observed for infectious and parasitic diseases, consistent with Al-Robai et al. (2022), who reported high ICF values for medicinal plants used to treat viral, fungal, bacterial, and other parasitic infections.

High ICF values often indicate that plants have potentially effective therapeutic properties (Heinrich et al., 1998). These findings suggest that the local community has a well-established and shared knowledge base regarding the therapeutic efficacy of certain medicinal plants, particularly for circulatory and blood-related conditions. This consensus not only helps prioritize these plants for further phytochemical and pharmacological investigations but also highlights their potential role in culturally relevant healthcare interventions.

### Direct matrix ranking

The DMR exercise was instrumental in identifying the most heavily used multipurpose plant species in the Metema district as well as the key threats to their sustainability. The three most utilized species were Z*iziphus spina-christi*, *X. americana,* and *F. sycomorus*, which are predominantly harvested for their medicinal properties rather than for non-medicinal uses. This finding aligns with previous research (Alemneh, 2021), which indicates that medicinal use is the primary threat to many plant species among the eight threats assessed in the DMR exercise.

The specific parts of these plants that are used for medicinal purposes significantly influence their vulnerability. For example, the root, root bark, and stem bark of *Z. spina-christi* have been harvested to treat various ailments, thereby increasing the susceptibility of plants to overexploitation. The frequent use of *X. americana* stem bark for medicinal purposes places additional pressure on this species. The DMR findings suggest that overharvesting of these multipurpose plants for medicinal applications poses a considerable threat to their long-term sustainability in the Metema district. These insights underscore the need for conservation strategies that balance the medicinal and non-medicinal uses of valuable plant resources.

### Novel ethnobotanical findings

Of the 85 medicinal plant species identified in this study, 79 had already been reported as traditional medicinal plants in Ethiopia, although the plant parts used, ailments treated, and mode of remedy preparation may vary from area to area as well as culture to culture. This high overlap suggests a strong cultural exchange of plant-based healthcare knowledge among the Ethiopian communities (Agize et al., 2022). However, six species have been documented for the first time as medicinal plants in Ethiopia, highlighting the biodiversity and ethnobotanical richness of the study area. To the best of our knowledge, *the medicinal use of T. venosa has not been reported elsewhere*, marking a novel discovery on a global scale. The identification of this plant as a new medicinal species highlights the unique health practices of the Metema district, including the use of plants unrecognized for their therapeutic potential elsewhere.

The findings of this study expand the understanding of traditional plant-based medicine in Metema, achieved through an ethnically focused approach and exploration of less studied areas. Similarly, other studies (Subba et al., 2016) have shown that studying under-explored ethnic communities can reveal new medicinal plant species. The discovery of these previously undocumented medicinal plants in Metema highlights the importance of the continued documentation of traditional ethnobotanical knowledge in Ethiopia. Further phytochemical research on these newly reported plants and their uses is essential to validate their medicinal efficacies. This line of research has the potential to contribute valuable insights to medical sciences (Iwu, 2002) while strengthening traditional healthcare practices passed down through generations (Subba et al., 2016).

### Threats to medicinal plants and conservation efforts

The primary threats to medicinal plants in the Metema district include the use of fuelwood, agricultural land expansion, and the application of herbicides and insecticides, consistent with findings from previous research in Ethiopia (Zemede et al., 2024). Additional threats identified include the use of pant parts for construction, human-induced fires, and overgrazing. The unsustainable exploitation of specific species such as *X. americana*, *C. spinarum*, *S. longepedunculata*, and *B. papyrifera* further jeopardizes their survival. To mitigate these threats, it is essential to implement awareness programs and conservation education programs that engage stakeholders and local communities. However, currently, the study area has limited conservation and management practices for medicinal plants. Reported local conservation efforts include cultivating medicinal plants in home gardens and preserving them on agricultural lands.

In addition to their medicinal uses, the plant species documented in this study serve multiple vital purposes in local communities. They are cultivated for their aesthetic value, are used as living fences, and provide food, shade, and fuelwood. This diverse range of uses underscores the cultural and ecological significance of natural resources. Strengthening the conservation and management practices surrounding these medicinal plants is essential to ensure their long-term sustainability and continued availability to the local population. Protecting these invaluable natural resources is essential not only for preserving traditional medicine knowledge and practices but also for enhancing food security within the area.

## Conclusion

The ethnobotanical study conducted in Metema district has yielded valuable insights into the intricate relationship between local communities and their use of medicinal plants. The findings revealed that knowledge of medicinal plants is predominantly held by older male informants, indicating a concerning trend: traditional knowledge is at risk of erosion as the older generation passes away without adequately transferring this wisdom to younger community members. This trend highlights the urgent need for strategies to preserve and document indigenous knowledge before it can be lost.

This study identified and documented 85 medicinal plant species across the three ethnic groups, emphasizing the rich biodiversity present in the study area. Notably, the Fabaceae family was the most represented among medicinal plants, reflecting their adaptability and ecological significance. The predominant use of leaves for medicinal preparations demonstrates a sustainable approach that minimizes the impact on plant populations. However, there are concerns about the overharvesting of roots, which could threaten the long-term survival of some species.

Moreover, the findings suggest that a considerable portion of medicinal plants are utilized to treat various ailments, indicating the community’s deep reliance on these resources for healthcare. The identification of new medicinal plants, including *T. venosa*, shows the potential for further exploration of ethnobotanical diversity. However, the study also identified significant threats to medicinal plants, primarily due to unsustainable practices, such as fuelwood collection and agricultural expansion. Efforts to conserve these plants and associated indigenous knowledge are currently limited.

To address these challenges, awareness programs should be developed to educate local communities on the importance of conserving medicinal plants and the risks associated with overharvesting. Involving community stakeholders in conservation efforts can foster a sense of ownership and responsibility toward local resources. Promoting sustainable harvesting techniques for medicinal plants, particularly those with a high demand for roots and other vulnerable parts, is also essential.

Furthermore, phytochemical and pharmacological studies are recommended to validate the medicinal properties of the newly identified plants. Supporting and expanding local conservation practices, such as cultivating medicinal plants in home gardens, could also play a crucial role. By implementing these recommendations, the local community can safeguard its rich ethnobotanical heritage, while ensuring the sustainability of medicinal plant resources for future generations.

## Data Availability

The original contributions presented in the study are included in the article/Supplementary Material, further inquiries can be directed to the corresponding author.

## References

[B1] Abera B. (2014). Medicinal plants used in traditional medicine by Oromo people, Ghimbi District, Southwest Ethiopia. J. Ethnobiol. Ethnomed. 10, 40. 10.1186/1746-4269-10-40 24885586 PMC4060869

[B2] Adamu H. Bekele T. Dalle G. (2012). Floristic diversity, regeneration status, and vegetation structure of woodlands in Metema area, Amhara national regional state, northwestern Ethiopia. J. For. Res. 23, 391–398. 10.1007/s11676-012-0275-z

[B3] Adrien K. M. Guillaume S. K. H. Souleymane M. Lucien B. G. N’Guessan J. D. (2018). *In vitro* antibacterial and antidiarraheic activity of root bark extract of Anogeissus leiocarpa (Combretaceae) during an experimental bacterial diarrhea induced by *Escherichia coli* extended-spectrum-lactamases (ESBL) in albino Wistar rats. J. Med. Plants Res. 12 (27), 463–473. 10.5897/jmpr2018.6663

[B4] Adzu B. Haruna A. K. Salawu O. A. Katsayal U. D. Njan A. (2007). *In vivo* antiplasmodial activity of ZS-2A: a fraction from chloroform extract of Zizyphus spina-christi root bark against Plasmodium berghei berghei in mice. Int. J. Biol. Chem. Sci. 1 (3), 281–286. 10.4314/ijbcs.v1i3.39714

[B5] Agize M. Asfaw Z. Nemomissa S. Gebre T. (2022). Ethnobotany of traditional medicinal plants and associated indigenous knowledge in Dawuro Zone of Southwestern Ethiopia. J. Ethnobiol. Ethnomed. 18, 48. 10.1186/s13002-022-00546-4 35729583 PMC9210772

[B6] Ahmad M. Sultana S. Fazl-i-Hadi S. ben Hadda T. Rashid S. Zafar M. (2014). An ethnobotanical study of medicinal plants in high mountainous region of chail valley (district Swat- Pakistan). J. Ethnobiol. Ethnomed. 10, 36. 10.1186/1746-4269-10-36 24739524 PMC4022037

[B7] Akbulut S. Karakose M. Özkan Z. C. (2019). Traditional uses of some wild plants in Kale and Acıpayam provinces in Denizli. Kastamonu Univ. J. For. Fac. 19 (1), 72–81. 10.17475/kastorman.543529

[B8] Akbulut S. Karaköse M. Şen G. (2022). Medicinal plants used in folk medicine of akcaabat district (Turkey). Fresenius Environ. Bull. 31, 7160–7176.

[B9] Alemneh D. (2021). Ethnobotanical study of plants used for human ailments in yilmana densa and quarit districts of west gojjam zone, Amhara region, Ethiopia. BioMed Res. Int. 1, 6615666. 10.1155/2021/6615666 PMC7910050 33681356

[B10] Alemu M. Asfaw Z. Lulekal E. Warkineh B. Debella A. Sisay B. (2024). Ethnobotanical study of traditional medicinal plants used by the local people in Habru District, North Wollo Zone, Ethiopia. J. Ethnobiol. Ethnomed. 20, 4. 10.1186/s13002-023-00644-x 38178202 PMC10768247

[B11] Alexiades M. N. (1996). Selected guidelines for ethnobotanical research: a field manual. Bronx, New York, U.S.A: The New York Botanical Garden.

[B12] Al-Robai S. A. Ahmed A. A. E. Mohamed H. A. Ahmed A. A. Zabin S. A. Alghamdi A. A. A. (2022). Qualitative and quantitative ethnobotanical survey in Al baha province, southwestern Saudi Arabia. Diversity 14, 867. 10.3390/d14100867

[B13] Amjad M. S. Zahoor U. Bussmann R. W. Altaf M. Gardazi S. M. H. Abbasi A. M. (2020). Ethnobotanical survey of the medicinal flora of Harighal, Azad Jammu and Kashmir, Pakistan. J. Ethnobiol. Ethnomed. 16, 65. 10.1186/s13002-020-00417-w 33109243 PMC7590686

[B14] Amsalu N. Bezie Y. Fentahun M. Alemayehu A. Amsalu G. (2018). Use and conservation of medicinal plants by indigenous people of Gozamin Wereda, East Gojjam zone of Amhara region, Ethiopia: an ethnobotanical approach. Evidence-Based Complementary Altern. Med. 1, 2973513. 10.1155/2018/2973513 PMC5884302 29743921

[B15] Anwar T. Qureshi H. Sarwar G. Siddiqi E. H. Ahmad A. (2024). Exploring ethnomedicinal plants for primary health care needs in rural communities. Ecol. Front. 44 (6), 1187–1196. 10.1016/j.ecofro.2024.06.003

[B16] Ashagre M. Lulekal E. (2021). Cultural significance of medicinal plants in treating different human ailments in guji semi-pastoralist people, suro barguda district, west guji zone, oromia regional state, Ethiopia. J. Ethnobiol. Ethnomed. 17, 61. 10.1186/s13002-021-00487-4 34663365 PMC8524801

[B17] Atnafu H. Awas T. Alemu S. Wube S. (2018). Ethnobotanical study of medicinal plants in selale mountain ridges, North Shoa, Ethiopia. Int. J. Biodivers. 2 (6), 567–577. 10.15406/bij.2018.02.00114

[B18] Baldé A. M. Traoré M. S. Diallo M. S. T. Vlietinck A. Pieters L. (2015). Ethnobotanical survey, antimicrobial and anticomplement activities of Guinean medicinal plants traditionally used in the treatment of inflammatory diseases in Conakry and Dubreka. J. Plant Sci. 3 (1/2), 11–19. 10.11648/J.JPS.S.2015030102.13

[B19] Bartlett J. E. Kotrlik J. W. Higgins C. C. (2001). Organizational research: determining organizational research: determining appropriate sample size in survey research appropriate sample size in survey research. Inf. Technol., Learn. Performance J. 19 (1), 43–50.

[B20] Bhandari P. Gurung M. B. Subedi C. K. Chaudhary R. P. Basnet K. Gurung J. (2021). Traditional use of medicinal plants in the chyangthapu-phalaicha biological sub-corridor, panchthar district, kangchenjunga landscape, Nepal. Ethnobot. Res. Appl. 22, 1–43. 10.32859/era.22.25.1-43

[B21] Braide W. Dokubo K. O. Adeleye S. A. Uzoh C. V. Akobundu C. I. (2018). Phytochemical properties, toxicological screening and antibacterial qualities of various parts extracts of Ficus sycomorus. J. Med. Plant Herb. Ther. Res. 6, 1–8.

[B22] Bukatuka F. Ngombe K. Mutwale K. Moni B. Makengo K. Pambu L. (2016). Bioactivity and nutritional values of some Dioscorea species traditionally used as medicinal foods in bandundu, DR Congo. DR Congo. Eur. J. Med. Plants. 14 (1), 1–11. 10.9734/ejmp/2016/25124

[B23] Caniago I. Siebert S. (1998). Medicinal plants ecology, knowledge and conservation in Kalimantan, Indonesia. Econ. Bot. 52, 229–250. 10.1007/BF02862141

[B24] Castro Souza J. D. Estevão L. R. D. M. Baratella-Evêncio L. Vieira M. G. F. Simões R. S. Florencio-Silva R. (2017). Mast cell concentration and skin wound contraction in rats treated with *Ximenia americana* L. Acta Cir. Bras. 32 (2), 148–156. 10.1590/s0102-865020170207 28300877

[B25] Chekole G. (2017). Ethnobotanical study of medicinal plants used against human ailments in Gubalafto District, Northern Ethiopia. J. Ethnobiol. Ethnomed. 13, 55. 10.1186/s13002-017-0182-7 28978342 PMC5628421

[B26] Chekole G. Asfaw Z. Kelbessa E. (2015). Ethnobotanical study of medicinal plants in the environs of Tara-gedam and Amba remnant forests of Libo Kemkem District, northwest Ethiopia. J. Ethnobiol. Ethnomed. 11, 4. 10.1186/1746-4269-11-4 25572933 PMC4417315

[B27] Cheng Z. Hu X. Lu X. Fang Q. Meng Y. Long C. (2022). Medicinal plants and fungi traditionally used by Dulong people in Northwest Yunnan, China. Front. Pharmacol. 13, 895129. 10.3389/fphar.2022.895129 35614945 PMC9124798

[B28] Cordero C. S. Meve U. Alejandro G. J. D. (2022). Ethnobotanical documentation of medicinal plants used by the indigenous panay bukidnon in lambunao, iloilo, Philippines. Front. Pharmacol. 12, 790567. 10.3389/fphar.2021.790567 35082673 PMC8784692

[B29] Cotton C. M. (1996). Ethnobotany: principles and applications. New York, U.S.A: John Wiley and Sons.

[B30] CSA (2007). The 2007 population and housing census of Ethiopia. Addis Ababa. Fed. Democr. Repub. Ethiop. Popul. Census Comm.

[B31] Demie G. Negash M. Awas T. (2018). Ethnobotanical study of medicinal plants used by indigenous people in and around Dirre Sheikh Hussein heritage site of South-eastern Ethiopia. J. Ethnopharmacol. 220, 87–93. 10.1016/j.jep.2018.03.033 29601979

[B32] Eshete A. Sterck F. Bongers F. (2011). Diversity and production of Ethiopian dry woodlands explained by climate-and soil-stress gradients. For. Ecol. Manag. 261 (9), 1499–1509. 10.1016/j.foreco.2011.01.021

[B33] Faruque M. O. Feng G. Khan M. N. A. Barlow J. W. Ankhi U. R. Hu S. (2019). Qualitative and quantitative ethnobotanical study of the pangkhua community in bilaichari upazilla, rangamati district, Bangladesh. J. Ethnobiol. Ethnomed. 15, 8. 10.1186/s13002-019-0287-2 30722779 PMC6364474

[B34] Friis I. Demissew S. van Breugel P. (2010). Atlas of the potential vegetation of Ethiopia. Copenhagen, Denmark: The Royal Danish Academy of Sciences and Letters.

[B35] Gedif T. Hahn H. (2003). The use of medicinal plants in self-care in rural central Ethiopia. J. Ethnopharmacol. 87, 155–161. 10.1016/s0378-8741(03)00109-0 12860301

[B36] Getaneh Z. A. Demissew S. Woldu Z. Aynekulu E. (2023). Determinants of plant community along environmental gradients in Geramo forest, the western escarpment of the rift valley of Ethiopia. Plos one 18 (11), e0294324. 10.1371/journal.pone.0294324 38011089 PMC10681247

[B37] Giday M. Teklehaymanot T. Animut A. Mekonnen Y. (2007). Medicinal plants of the shinasha, agew-awi and Amhara peoples in northwest Ethiopia. J. Ethnopharmacol. 110, 516–525. 10.1016/j.jep.2006.10.011 17101251

[B38] Güler O. Polat R. Karaköse M. Çakılcıoğlu U. Akbulut S. (2021). An ethnoveterinary study on plants used for the treatment of livestock diseases in the province of Giresun (Turkey). South Afr. J. Bot. 142, 53–62. 10.1016/j.sajb.2021.06.003

[B39] Haq S. M. Hassan M. Bussmann R. W. Calixto E. S. Rahman I. U. Sakhi S. (2022). A cross-cultural analysis of plant resources among five ethnic groups in the Western Himalayan region of Jammu and Kashmir. Biology 11 (4), 491. 10.3390/biology11040491 35453691 PMC9032642

[B40] Hedberg I. Persson E. Edwards S. Demissew S. Kelbessa E. (2006). “Flora of Ethiopia and Eritrea,” in Gentianaceae to cyclocheilaceae, Vol. 5. Addis Ababa, Ethiopia; Uppsala, Sweeden: National Herbarium, Addis Ababa University.

[B41] Heinrich M. Ankli A. Frei B. Weimann C. Sticher O. (1998). Medicinal plants in Mexico: healers’ consensus and cultural importance. Soc. Sci. Med. 47 (11), 1859–1871. 10.1016/s0277-9536(98)00181-6 9877354

[B42] Heinrich M. Edwards S. Moerman D. E. Leonti M. (2009). Ethnopharmacological field studies: a critical assessment of their conceptual basis and methods. J. Ethnopharmacol. 124 (1), 1–17. 10.1016/j.jep.2009.03.043 19537298

[B43] Ishtiaq M. Mahmood A. Maqbool M. (2015). Indigenous knowledge of medicinal plants from Sudhanoti district (AJK) Pakistan. J. Ethnopharmacol. 168, 201–207. 10.1016/j.jep.2015.01.054 25666425

[B44] Iwu M. M. (2002). Ethnobotanical approach to pharmaceutical drug discovery: strengths and limitations. Adv. Phytomedicine 1, 309–320. 10.1016/s1572-557x(02)80034-4

[B45] Karaköse M. (2022). An ethnobotanical study of medicinal plants in Güce district, north-eastern Turkey. Plant Divers. 44 (6), 577–597. 10.1016/j.pld.2022.03.005 36540712 PMC9751085

[B46] Kefalew A. Asfaw Z. Kelbessa E. (2015). Ethnobotany of medicinal plants in ada’a district, east shewa zone of oromia regional state, Ethiopia. J. Ethnobiol. Ethnomed. 11, 25. 10.1186/s13002-015-0014-6 25889311 PMC4419563

[B47] Kidane L. Gebremedhin G. Beyene T. (2018). Ethnobotanical study of medicinal plants in ganta afeshum district, eastern zone of tigray, northern Ethiopia. J. Ethnobiol. Ethnomed. 14, 64. 10.1186/s13002-018-0266-z 30390675 PMC6215673

[B48] Kuniyal C. P. Bisht V. K. Negi J. S. Bhatt V. P. Bisht D. S. Butola J. S. (2015). Progress and prospect in the integrated development of medicinal and aromatic plants (MAPs) sector in Uttarakhand, Western Himalaya. Environ. Dev. Sustain. 17, 1141–1162. 10.1007/s10668-014-9595-9

[B49] Li F. S. Weng J. K. (2017). Demystifying traditional herbal medicine with modern approach. Nat. Plants 3 (8), 17109–17117. 10.1038/nplants.2017.109 28758992

[B50] Lulekal E. Asfaw Z. Kelbessa E. Damme P. V. (2013). Ethnomedicinal study of plants used for human ailments in ankober district, north shewa zone, Amhara region, Ethiopia. J. Ethnobiol. Ethnomed. 9, 63. 10.1186/1746-4269-9-63 23984919 PMC3846447

[B51] Magalhães K. N. Guarniz W. A. S. Sá K. M. Freire A. B. Monteiro M. P. Nojosa R. T. (2019). Medicinal plants of the Caatinga, northeastern Brazil: Ethnopharmacopeia (1980–1990) of the late professor Francisco José de Abreu Matos. J. Ethnopharmacol. 237, 314–353. 10.1016/j.jep.2019.03.032 30885881

[B52] Mahomoodally M. F. (2013). Traditional medicines in Africa: an appraisal of ten potent African medicinal plants. Evidence-Based Complementary Altern. Med. 1, 617459. 10.1155/2013/617459 PMC3866779 24367388

[B53] Mamun A. A. Khan M. S. S. (2020). A review of significance of herbal medicine and its evolution as a therapeutics in global healthcare. Aust. Herb. Insight 3 (1), 1–9. 10.25163/ahi.3121063

[B54] Martin G. J. (1995). Ethnobotany: a methods manual. London, U.K: Chapman and Hall.

[B55] Martin G. J. (2004). Ethnobotany: a methods manual. London, U.K: Earthscan.

[B56] Masresha G. Melkamu Y. Walle G. C. (2023). Ethnobotanical study on wild edible plants in Metema district, Amhara regional state, Ethiopia. Int. J. For. Res. 1, 9243343. 10.1155/2023/9243343

[B57] Masters E. T. (2023). Medicinal plants of the upper Aswa River catchment of northern Uganda - a cultural crossroads. J. Ethnobiol. Ethnomed. 19, 48. 10.1186/s13002-023-00620-5 37884931 PMC10605377

[B58] Mekuria T. Abduro H. (2022). Ethnobotanical study on medicinal plants used by local communities in shashemene district, west arsi zone of oromia region, Ethiopia. Asian J. Plant Soil Sci. 7 (1), 158–170. 10.56557/ajopss/2022/v7i173

[B59] Mengistu M. Abebe Y. Mekonnen Y. Tolessa T. (2012). *In vivo* and *in vitro* hypotensive effect of aqueous extract of Moringa stenopetala. Afr. Health Sci. 12 (4), 545–551. 10.4314/ahs.v12i4.23 23515422 PMC3598298

[B60] Muluye A. B. Ayicheh M. W. (2020). Medicinal plants utilized for hepatic disorders in Ethiopian traditional medical practices: a review. Int. J. Phytomed. Phytother. 6, 52. 10.1186/s40816-020-00195-8

[B61] Mzena T. Swai H. Chacha M. (2018). Antimalarial activity of *Cucumis metuliferus* and *Lippia kituiensis* against *Plasmodium berghei* infection in mice. Res. Rep. Trop. Med. 9, 81–88. 10.2147/RRTM.S150091 30050358 PMC6049058

[B62] Nigussie B. Kim Y.-D. (2019). Ethnobotanical study of medicinal plants in the Hawassa Zuria district, sidama zone, southern Ethiopia. J. Ethnobiol. Ethnomed. 15, 25. 10.1186/s13002-019-0302-7 31126296 PMC6534827

[B63] Nwinyi F. C. Abdu P. Saidu S. N. Omamegbe J. Mohammed A. (2021). Pharmacological justification for the ethnomedicinal use of stem bark extract of *Stereospermum kunthianum* Cham.(Family: bignoniaceae) for wound healing effects. J. Med. Plants 9 (2), 19–27.

[B64] Ojha S. N. Tiwari D. Anand A. Sundriyal R. C. (2020). Ethnomedicinal knowledge of a marginal hill community of Central Himalaya: diversity, usage pattern, and conservation concerns. J. Ethnobiol. Ethnomed. 16, 29. 10.1186/s13002-020-00381-5 32448334 PMC7245762

[B65] Oseni L. A. Akwetey G. M. (2012). An *in-vivo* evaluation of antiplasmodial activity of aqueous and ethanolic leaf extracts of *Azadirachta indica* in *Plasmodium berghei* infected balb/c mice. Int. J. Pharm. Sci. Res. 3 (5), 1406–1410.

[B66] Pala N. A. Sarkar B. C. Shukla G. Chettri N. Deb S. Bhat J. A. (2019). Floristic composition and utilization of ethnomedicinal plant species in home gardens of the Eastern Himalaya. J. Ethnobiol. Ethnomed. 15, 14. 10.1186/s13002-019-0293-4 30782184 PMC6380006

[B67] Ralte L. Sailo H. Singh T. (2024). Ethnobotanical study of medicinal plants used by the indigenous community of the western region of Mizoram, India. J. Ethnobiol. Ethnomed. 20, 2. 10.1186/s13002-023-00642-z 38172927 PMC10765666

[B68] Regassa R. (2013). Assessment of indigenous knowledge of medicinal plant practice and mode of service delivery in Hawassa city, southern Ethiopia. J. Med. Plants Res. 7 (9), 517–535.

[B69] Regassa R. Bekele T. Megersa M. (2017). Ethnobotanical study of traditional medicinal plants used to treat human ailments by halaba people, southern Ethiopia. J. Med. Plants Stud. 5 (4), 36–47.

[B70] Sanwal R. Chaudhary A. K. (2011). Wound healing and antimicrobial potential of Carissa spinarum Linn. in albino mice. J. Ethnopharmacol. 135 (3), 792–796. 10.1016/j.jep.2011.04.025 21527332

[B71] Şen G. Akbulut S. Karaköse M. (2022). Ethnopharmacological study of medicinal plants in Kastamonu province (Türkiye). Open Chem. 20 (1), 873–911. 10.1515/chem-2022-0204

[B72] Shakya A. K. (2016). Medicinal plants: future source of new drugs. Int. J. Herb. Med. 4 (4), 59–64.

[B73] Sop T. K. Oldeland J. Bognounou F. Schmiedel U. Thiombiano A. (2012). Ethnobotanical knowledge and valuation of woody plants species: a comparative analysis of three ethnic groups from the sub-Sahel of Burkina Faso. Environ. Dev. Sustain. 14 (5), 627–649. 10.1007/s10668-012-9345-9

[B74] Subba B. Srivastav C. Kandel R. C. (2016). Scientific validation of medicinal plants used by Yakkha community of Chanuwa VDC, Dhankuta, Nepal. SpringerPlus 5, 155. 10.1186/s40064-016-1821-5 27026852 PMC4766171

[B75] Tadesse D. Masresha G. Lulekal E. (2024). Ethnobotanical study of medicinal plants used to treat human ailments in Quara district, northwestern Ethiopia. J. Ethnobiol. Ethnomed. 20, 75. 10.1186/s13002-024-00712-w 39127690 PMC11317005

[B76] Tadeyos M. Wendawek A. (2022). An ethnobotanical study of medicinal plants in dilla zuria woreda of gedo zone southern Ethiopia. Glob. J. Ecol. 7 (11), 001–012. 10.17352/gje.000053

[B77] Tahir M. Asnake H. Beyene T. Damme P. V. Mohammed A. (2023). Ethnobotanical study of medicinal plants in asagirt district, northeastern Ethiopia. Trop. Med. Health 51, 1. 10.1186/s41182-023-00493-0 36617576 PMC9827656

[B78] Tahir M. Gebremichael L. Beyene T. Damme P. V. (2021). Ethnobotanical study of medicinal plants in adwa district, central zone of tigray regional state, northern Ethiopia. J. Ethnobiol. Ethnomed. 17, 71. 10.1186/s13002-021-00498-1 34952609 PMC8709991

[B79] Tamene S. Negash M. Makonda F. B. Chiwona-Karltun L. (2024). Influence of socio-demographic factors on medicinal plant knowledge among three selected ethnic groups in south-central Ethiopia. J. Ethnobiol. Ethnomed. 20, 29. 10.1186/s13002-024-00672-1 38419117 PMC11340053

[B80] Tefera B. N. Kim Y.-D. (2019). Ethnobotanical study of medicinal plants in the hawassa zuria district, sidama zone, southern Ethiopia. Ethnobiol. Ethnomed. 15, 25. 10.1186/s13002-019-0302-7 PMC6534827 31126296

[B81] Teka A. Asfaw Z. Demissew S. Damme P. V. (2020). Medicinal plant use practice in four ethnic communities (Gurage, Mareqo, Qebena, and Silti), south central Ethiopia. J. Ethnobiol. Ethnomed. 16, 27. 10.1186/s13002-020-00377-1 32448337 PMC7245860

[B82] Teklehaymanot T. Giday M. (2010). Quantitative ethnobotany of medicinal plants used by kara and kwego semi-pastoralist people in lower omo river valley, debub omo zone, southern nations, nationalities and peoples regional state, Ethiopia. J. Ethnopharmacol. 130 (1), 76–84. 10.1016/j.jep.2010.04.013 20420888

[B83] Tuasha N. Petros B. Asfaw Z. (2018). Plants used as anticancer agents in the Ethio pian traditional medical practices: a systematic review. Evidence-based Complementary Altern. Med. 1, 6274021. 10.1155/2018/6274021 PMC6192134 30402131

[B84] Tugume P. Kakudidi E. K. Buyinza M. Namaalwa J. Kamatenesi M. Mucunguzi P. (2016). Ethnobotanical survey of medicinal plant species used by communities around Mabira Central Forest Reserve Uganda. J. Ethnobiol. Ethnomed. 12, 5. 10.1186/s13002-015-0077-4 26762159 PMC4712608

[B85] Tuttolomondo T. Licata M. Leto C. Savo V. Bonsangue G. Letizia Gargano M. (2014). Ethnobotanical investigation on wild medicinal plants in the monti sicani regional park (sicily, Italy). J. Ethnopharmacol. 153 (3), 568–586. 10.1016/j.jep.2014.02.032 24632020

[B86] WHO (2023). ICD-11 International Classification of Diseases for mortality and morbidity statistics. Version: 01/2023.

[B87] Wiryono W. Wanandi Y. Ilahi A. K. Deselina D. Senoaji G. Siswahyono S. (2019). The local knowledge of the plant names and uses by Semende tribe people in Kaur district Bengkulu province Indonesia. Biodiversitas 20 (3), 754–761. 10.13057/biodiv/d200320

[B88] Yimam M. Yimer S. M. Beressa T. B. (2022). Ethnobotanical study of medicinal plants used in artuma fursi district, Amhara regional state, Ethiopia. Trop. Med. Health 50, 85. 10.1186/s41182-022-00438-z 36352467 PMC9644516

[B89] Zemede J. Mekuria T. Ochieng C. O. Onjalalaina G. E. Hu G. W. (2024). Ethnobotanical study of traditional medicinal plants used by the local Gamo people in Boreda Abaya District, Gamo Zone, southern Ethiopia. J. Ethnobiol. Ethnomed. 20, 28. 10.1186/s13002-024-00666-z 38419092 PMC10900619

