# Supplementary material for: Ethnopharmacological study of traditional medicinal plants used by the people in Metema district, northwestern Ethiopia
Source: Front Pharmacol. 2025 Mar 10;16:1535822. doi: 10.3389/fphar.2025.1535822 (PMC11931033; doi:10.3389/fphar.2025.1535822)
Supplement: Supplementary file 1 [file Table1.docx]

Supplementary table 1. List of medicinal and magical plant species used by the communities of the Metema district, Northwestern Ethiopia

| **Family** | **Scientific name** | **Local name**  **(A, Ag, G)** | **H** | **PU** | **CPU** | **Ailment treated** | **Methods of Preparation and Application** | **RA** | **Vouch. No.** |
| --- | --- | --- | --- | --- | --- | --- | --- | --- | --- |
| Amaranthaceae | *Amaranthus caudatus* L. | Aluma large seed (A) | H | L | F | Heart disease | Cook as stew and eat | O | DTK21 |
|  |  |  |  | L | F | Jaundice | Cook as stew and eat | O |  |
|  |  |  |  | S | F | Coughing | Roast, grind, boil, add water and drink | O |  |
|  | *Amaranthus hybridus* L. | Aluma small seed (A) | H | L | F | Constipation | Cook as stew and eat | O | DTK20 |
| Amaryllidaceae | *Allium cepa* L. | Qey Shinkurt (A) | H | Bu | F | Hypertension | Grind, put into water, and drink the filtrate | O | DTK40 |
|  | *Allium sativum* L. | Nech Shinkurt (A) | H | Bu | F/D | Common cold | Crush, and eat the raw by mixing it with honey or insert inside the nose | O/T | DTK34 |
|  |  |  |  | Bu | F/D | Stomachache | Eat the raw | O |  |
|  | *Crinum abyssinicum* Hochst. ex A. Rich. | Yejib Shinkurt (A) | H | Bu | F/D | Bacterial infection on the tip of a finger | Crush and tie | T | DTK118 |
|  |  |  |  | Bu | F/D | Snakebite | Crush and tie | T |  |
|  |  |  |  | Bu | F/D | Scorpion sting | Crush and tie | T |  |
| [Apocynaceae](https://powo.science.kew.org/taxon/urn:lsid:ipni.org:names:30000008-2) | *Calotropis procera* (Aiton) W.T. Aiton | Tobia (A)  Tebaya (Ag)  Papeziwta (G) | S | La | F | Wound healing | Paint the fluid | T | DTK116 |
|  |  |  |  | La | F | Ringworm | Paint the fluid | T |  |
|  |  |  |  | La | F | Bacterial infection on the tip of a finger | Paint and tie | T |  |
|  |  |  |  | La | F | Circumcision wound | Paint the fluid | T |  |
|  |  |  |  | R | F | Gum disease | Hold with teeth | O |  |
|  |  |  |  | R | F | Cancer | Burn gently and touch the area | T |  |
|  |  |  |  | L | F | Cancer | Burn gently and touch the area | T |  |
|  |  |  |  | La | F | Cancer | Paint the fluid | T |  |
|  |  |  |  | La | F | Wart | Paint the fluid | T |  |
|  | *Carissa spinarum* L. | Agam (A) | S | R | F/D | Sudden sickness | Inhale the smoke that has been placed on the fire | I | DTK83 |
|  |  |  |  | R | F/D | Headache | Inhale the smoke that has been placed on the fire | I |  |
|  |  |  |  | R | F | Snakebite | Crush it with the roots of *Ruta chalepensis* and the bulb of *Allium sativum*, add water, and drink | O |  |
|  |  |  |  | R | F | Evil eye | Crush with the bulb of *Allium sativum* and sniff or drink by mixing with water | I/O |  |
|  | *Leptadenia arborea* (Forssk.) Schweinf. | Shaloba (G) | C | R | F/D | Uterus sore | Crush, mix with water and boil, and then wash the vagina when cool | T | DTK51 |
|  | *Tacazzea venosa* Decne. | Arut (A) | S | R | F | Evil spirit | Pound by mixing with the roots of *Withania somnifera, Sida rhombifolia*, *Securidaca longepedunculata,* and *Clerodendrum alatum* and put on fire and fumigate | I | DTK158 |
| Bignoniaceae | *Stereospermum kunthianum* Cham. | Zana (A)  Arizane (Ag)  Andigila (G) | T | SB | F | Bleeding gum | Brush the teeth | O | DTK112 |
|  |  |  |  | SB | F/D | Wound healing | Crush and paste | T |  |
|  |  |  |  | SB | F/D | Snakebite | Crush and paste | T |  |
| Boraginaceae | *Cordia africana* Lam. | Wanza (A)  Bagusie (Ag)  Banja (G) | T | L | F | Febrile illness | Boil with the leaves of *Moringa stenoptela*, wait until cool, and drink | O | DTK110 |
|  |  |  |  | L | F | Jaundice | Crush, mix with chick pea. Boil and eat | O |  |
|  |  |  |  | SB | F | Jaundice | Crush, boil the decoction, and drink before breakfast | O |  |
|  |  |  |  | SB | F | Bleeding wound | Crush and paste | T |  |
|  |  |  |  | R | F | Jaundice | Crush, mix with cold water and drink | O |  |
|  |  |  |  | SB | F | Jaundice | Crush, mix with cold water and drink | O |  |
|  |  |  |  | SB | F | Circumcision wound | Dump the removed part by covering it with the stem bark | - |  |
|  | *Trichodesma zeylanicum*(Burm.f.) R.Br. | Kuskuas (A) | H | L | F | Scabies | Squeeze and paste | T | DTK33 |
| Brassicaceae | *Lepidium sativum* L. | Feto (A) | H | S | D | Evil eye | Crush with the bulb of *Allium sativum* and smell | I | DTK39 |
|  |  |  |  | S | D | Tendinitis | Crush, mix with honey and drink | O |  |
|  | *Boswellia papyrifera* (Caill.) Hochst. | Walya Meker (A)  Golgola (G) | T | SB | D | Spider poison | Crush, mix with butter and paint | T | DTK87 |
|  |  |  |  | SB | F | Uvulitis | Crush, squeeze, and drink | O |  |
| Caricaceae | *Carica papaya* L. | Papaya (A) | T | L | F | Febrile illness | Boil the part by mixing it with the leaf of *Cordia africana* and then inhale | I | DTK125 |
|  |  |  |  | L | F | Stomach ache | Boil and drink the decoction when cool | O |  |
|  |  |  |  | L | F | Malaria | Boil and drink the decoction | O |  |
| Combretaceae | *Combretum aculeatum* Vent. | Forha (A)  Afu (G) | S | SB | D | Cancer | Crush, add milk and drink | O | DTK15 |
|  | *Combretum molle* R.Br. ex G. Don | Avalo (A)  Telmeseya (G) | T | SB | F | Jaundice | Boil with stem bark of *Cordia africana* and drink the decoction when cool | O | DTK113 |
|  |  |  |  | SB | F | Malaria | Boil with stem bark of *Cordia africana* and drink the decoction when cool | O |  |
|  | *Terminalia laxiflora* Engl. | Wonbela (A)  Bora (G) | T | R | F | Snakebite | Put the root tip on fire and put it on the bitten area | T | DTK72 |
|  |  |  |  | RB | F | Teething in babies | Brush the teeth | O |  |
|  | *Terminalia leiocarpa* (DC.) Baill. | Kekera (A)  Sigah (G) | T | SB | F/D | Uvulitis | Put the pieces to their head for a while and say “put up. Put up” | T | DTK73 |
|  |  |  |  | SB | F | Tape worm | Crush, mix with water and drink | O |  |
|  |  |  |  | SB | F | Dysuria | Crush, mix with water, boil, and drink after adding sugar | O |  |
|  |  |  |  | SB | F | Jaundice | Crush, put to the boiling *Cicer arietinum,* and drink the filtrate | O |  |
|  |  |  |  | SB | F | Amoebiasis | Crush, boil, and drink the decoction when cool | O |  |
| Commelinaceae | *Commelina imberbis* Ehrenb. ex Hassk. | Yewuha Enkur (A) | H | L | F | Scabies | Brush the affected body | T | DTK36 |
| Cucurbitaceae | *Cucumis ficifolius* A. Rich. | Yemdir Embuay (A) | H | R | F | Snakebite | Chew and take the sap | O | DTK78 |
|  |  |  |  | R | F/D | Jaundice | Crush with the roots of *Withania somnifera* and *Carissa spinarum* mix with water and drink | O |  |
| Cucurbitaceae | *Cucumis metuliferus* E. Mey. ex Naudin | Yemta tira (G) | H | F | F | Wound on penis | Paint with fruit jelly | T | DTK142 |
|  | *Cucumis melo* L. | Yayit duba (A) | H | R | F | Spider poison | Chew and spat | T | DTK143 |
|  |  |  |  | R | F | Scorpion sting | Chew and spat | T |  |
|  | *Cucurbita pepo* L. | Duba (A)  Patuwa (G) | H | L | F | Abdominal bloating | Put on burning fire gently and put it on the surface of the stomach | T | DTK127 |
|  |  |  |  | S | F/D | Tape worm | Roast, grind and eat with local bread called *injera* | O |  |
|  |  |  |  | L | F | Ear lesion | Squeeze the bud and drop | A |  |
|  | *Lagenaria siceraria* (Molina) Standley | Qil (A) | H | F | D | Uvulitis | Hang inside the house | - | DTK128 |
|  |  |  |  | L | F | Ear lesion | Squeeze bud and drop | A |  |
|  |  |  |  | F | D | Stabbing pain | Put on fire and gently touch | T |  |
|  |  |  |  | L | F | Snakebite | Crush, mix with water, and goat excrete and drink | O |  |
|  | *Momordica foetida* Schumach. | Yekura hareg (A)  Yabda (G) | H | L | F | Malaria | Boil the bud by mixing with salt and pepper and eat with local bread called *injera* | O | DTK08 |
|  |  |  |  | L | F | Amoebiasis | Boil the bud by mixing with salt and pepper and eat with local bread called *injera* | O |  |
|  | *Zehneria scabra* (L.f.) Sonder | Hareg resa (A) | H | L | F/D | Febrile illness | Squeeze, add to coffee and drink | O | DTK79 |
| Dioscoreaceae | *Dioscorea dumetorum* (Kunth) Pax | Chibalya (G) | C | T | F | Diarrhea | Boil and eat | O | DT145 |
|  |  |  |  | T | F | Diabetes mellitus | Boil and eat | O |  |
|  | *Dioscorea praehensilis* Benth. | Sinsa (A)  Yecha (G) | C | T | F/D | Rectal prolapse | Boil and eat | O | DTK12 |
|  |  |  |  | T | F | Diarrhea | Boil and eat | O |  |
| Ebenaceae | *Diospyros mespiliformis* Hochst. ex A. DC. | Serkin (A)  Serkuni (Ag)  Eitege (G) | T | F | F | Ringworm | Paint the affected area with the unripe fruit | T | DTK90 |
| Euphorbiaceae | *Jatropha curcas* L. | Awir (A) | T | S | D | Malaria | Crush, homogenize with cold water and drink | O | DTK104 |
| Fabaceae | *Faidherbia albida*(Delile) A. Chev. | Gorgoro (A)  Enguwa (G) | T | R | F/D | Evil eye | Put on fire and inhale | I | DTK56 |
|  |  |  |  | R | F | Spider poison | Burn on fire, mix with butter and paint | T |  |
|  |  |  |  | R | F | Malaria | Chew and take the sap | O |  |
|  |  |  |  | R | F | Osteoporosis | Pound and tie | T |  |
|  |  |  |  | G | F | Ear pest | Pour it through the ear | A |  |
|  | *Piliostigma thonningii* (Schumach.) Milne-Redh. | Yekola Wanza (A)  Dawdie (Ag)  Mecha (G) | T | R | F | Gastritis | Chew and take the fluid | O | DTK60 |
|  | *Pterocarpus lucens* Lepr. ex Guill. & Perr. | Charia (A) Chaya (G) | T | SB | F | Snakebite | Crush, mix with water and drink | O | DTK01 |
|  |  |  |  | SB | F | Rabies | Crush, collect the fluid, and drink with milk | O |  |
|  | *Tamarindus indica* L. | Kumer (A)  Chamie (Ag)  Degua (G) | T | F | F | Malaria | Dilute with water and drink | O | DTK61 |
|  |  |  |  | F | F/D | Abdominal bloating | Mix with water, sugar and drink | O |  |
|  | *Trigonella foenum-graecum* L. | Abish (A) | H | S | D | Spider poison | Roast, pound, dilute with water, and paint | T | DTK63 |
|  | *Vachellia sieberiana*(DC.) Kyal. & Boatwr. | Nech Girar (A)  Sipa (G) | T | R | D | Sudden sickness | Crush the part, boil it and inhale | I | DTK59 |
|  |  |  |  | R | F/D | Scorpion sting | Crush and tie | T |  |
| Lamiaceae | *Ocimum lamiifolium* Hochst. Ex Benth. | Dama Kessie (A) | S | R | F/D | Bleeding gum | Boil and wash the mouth | O | DTK120 |
|  |  |  |  | La | F | Bleeding wound | Paint the fluid | T |  |
| Linaceae | *Linum usitatissimum* L. | Telba (A) | H | S | D | Retained placenta | Roast, pound, boil the decoction, and drink when cool | O | DTK54 |
|  |  |  |  | S | D | Dirt on the eye | Chew and spat | Op |  |
|  |  |  |  | S | D | Constipation | Roast, pound, boil the decoction, and drink when cool | O |  |
| Malvaceae | *Abelmoschus esculentus* (L.) Moench | Wayka (A) | H | F | F | Back pain | Cook as stew and eat with local bread called *injera* | O | DTK85 |
|  |  |  |  | F | F | Gastritis | Cook as stew and eat with local bread called *injera* | O |  |
|  | *Abelmoschus ficulneus* (L.) Wight & Arn. | Yebereha wayka (A) | H | F | D | Kidney disease | Mix the powder with warm water and drink | O | DTK11 |
|  |  |  |  | S | F/D | Gastritis | Roast, pound, boil and drink like coffee | O |  |
|  |  |  |  | F | F/D | Constipation | Cook as stew and eat with local bread called *injera* | O |  |
|  | *Corchorus olitorius* L. | Kudra (A)  Lalqa (G) | H | L | F | Constipation | Prepare stew by adding all ingredients and eat with bread or local bread called *injera* | O | DTK25 |
|  |  |  |  | L | F | Back pain | Prepare stew by adding all ingredients and eat with bread or local bread called *injera* | O |  |
|  | *Gossypium hirsutum* L. | Tit (A) | S | SO | D | Gout | Crush and drink the oil | O | DTK45 |
|  |  |  |  | S | D | Bleeding wound | Pound and paste | T |  |
|  | *Grewia ferruginea* Hochst. ex A. Rich. | Lenquata (A)  Gedeya (G) | T | SB | F | Bleeding wound | Crush, homogenize with cold water, and tie | T | DTK109 |
|  |  |  |  | SB | F | Satisfy sexual desire | Mix with water, paint the tip of the penis, and rub repeatedly | T |  |
|  |  |  |  | SB | F | To remove lice | Mix with cold water and wash | T |  |
|  |  |  |  | L | F | Constipation | Prepare stew and eat | O |  |
|  |  |  |  | SB | F | Dandruff | Mix with cold water and wash | T |  |
|  | *Grewia flavescens* Juss. | Betre Mussie (A)  Anjishefro (G) | S | F | F | Anemia | Mix with cold water and drink the filtrate | O | DTK24 |
|  | *Sida rhombifolia*  var. *serratifolia* (R.Wilczek & Steyaert) | Gurjejit (A)  Birjeguana (G) | H | L | F | Bleeding wound | Squeeze and paste to the affected area | T | DTK09 |
|  |  |  |  | L | F | Ringworm | Squeeze and paste | T |  |
|  |  |  |  | L | f | Circumcision wound | Grind and paste | T |  |
|  |  |  |  | L | F | Wound healing | Squeeze, paste, and tie | T |  |
|  |  |  |  | L | F | Bacterial infection on the tip of a finger | Chew, paste, and tie | T |  |
|  | *Sterculia setigera* Delile | Darlie (A) Yabuwa (G) | T | F | F | Teething in babies | Brush the gum | O | DTK94 |
|  |  |  |  | Tw | F/D | Sudden sickness | Tie on the finger | T |  |
| Meliaceae | *Azadirachta indica* A. Juss. | Nim (A) | T | L | F | Malaria | Squeeze the buds, add water and drink | O | DTK23 |
|  |  |  |  | L | F | Hair loss | Squeeze the buds, paint | T |  |
|  |  |  |  | Tw | F | Bleeding gum | Brush the teeth | O |  |
|  |  |  |  | L | F | Febrile illness | Squeeze the buds, add water and drink | O |  |
|  |  |  |  | L | F | Jaundice | Squeeze the buds, add water and drink | O |  |
|  |  |  |  | L | F | Uvulitis | Squeeze the buds, add water and drink | O |  |
| Moraceae | *Adansonia digitata* L. | Diza (A)  Fertata (Ag)  Giya (G) | T | F | F/D | Intestinal parasite | Dilute with water and drink by adding salt or sugar | O | DTK64 |
|  |  |  |  | F | D | Male erectile dysfunction | Mix with honey and eat | O |  |
|  | *Ficus thonningii* Blume. | Chibha (A)  Asiyah (G) | T | LA | F | Spider poison | Paint the fluid | T | DTK66 |
|  |  |  |  | La | F | Wart | Drop by piercing it | T |  |
|  |  |  |  | L | F | Febrile illness | Boil the part by mixing it with the leaves of *Cordia africana* and inhale it | I |  |
|  | *Ficus sycomorus* L. | Bamba (A)  Bambibi (Ag)  Fuka (G) | T | L | F | Febrile illness | Boil by mixing with leaves of *Cordia africana* and *Zehneria scabra*, drink the filtrate as well as wash the whole body | O/T | DTK65 |
|  |  |  |  | L | F | Sudden sickness | Boil by mixing with leaves of Z*ehneria scabra*, and then wash the whole body | T |  |
|  |  |  |  | La | F | Wound healing | Paint the fluid | T |  |
|  |  |  |  | La | F | Spider poison | Paint the fluid | T |  |
|  |  |  |  | SB | F | Snakebite | Crush and paste | T |  |
|  | *Ficus vasta* Forssk. | Warka (A)  Afuqa (G) | T | L | F | Febrile illness | Boil and inhalation | I | DTK68 |
|  |  |  |  | SB | F | Gum disease | Chew and take the fluid | O |  |
| Moringaceae | *Moringa stenopetala* (Baker f.) Cufod. | Shiferaw (A) | T | L | F/D | Jaundice | Boil and drink the decoction as tea when cool | O | DTK29 |
|  |  |  |  | L | F | Gastritis | Boil and drink the decoction as tea when cool | O |  |
|  |  |  |  | L | F/D | Hypertension | Boil and drink the decoction as tea when cool or squeeze by adding a drop of water, collect in a coffee cup, and drink | O |  |
|  |  |  |  | L | F/D | Malaria | Boil and drink the decoction as tea when cool | O |  |
|  |  |  |  | Fl | F | Asthma | Boil and drink the decoction as tea when cool | O |  |
|  |  |  |  | L | F | Common cold | Boil and drink the decoction as tea when cool | O |  |
|  |  |  |  | L | F/D | Febrile illness | Boil with the leaves of *Zehneria scabra* and fumigate the body or boil the leaves, and drink them as tea by adding sugar | T/O |  |
|  |  |  |  | L | F/D | Diabetes mellitus | Boil and drink the decoction as tea when cool | O |  |
| Olacaceae | *Ximenia americana* L. | Enkoy (A)  Tatakuy (Ag)  Fiya (G) | S | SB | F/D | Bleeding wound | Crush and paste | T | DTK89 |
|  |  |  |  | SB | F/D | Eye infection | Crush, homogenize with warm water and fumigate | T |  |
|  |  |  |  | SB | F | Uvulitis | Crush, mix with water and drink | O |  |
|  |  |  |  | L | F | Uvulitis | Put the part on the head of the baby and say “Return it, return it back” repeatedly | T |  |
| Oleaceae | *Jasminum grandiflorum* L. | Tembelel (A) | C | R | F/D | Evil spirit | Crush, homogenize with cold water and wash the body | T | DTK93 |
| Pedaliaceae | *Sesamum indicum* L. | Selit (A) | H | S | D | Constipation | Roast, pound, and eat | O | DTK46 |
| Phytolaccaceae | *Phytolacca dodecandra* L'Hér. | Indod (A) | C | R | F/D | Rabies | Crush, homogenize with cold water, and drink | O | DTK121 |
| Poaceae | *Eleusine coracana* (L.) Gaertn*.* | Dagusa (A) | H | St | F | Back pain | Chew and take the fluid | O | DTK05 |
|  | *Eragrostis tef* (Zuccagni) Trotter | Tef (A) | H | S | D | Coughing | Grind, mix with hot water to prepare አጥሚት and drink | O | DTK132 |
|  |  |  |  | S | D | Back pain | Prepare as porridge and eat | O |  |
|  |  |  |  | S | D | Tonsillitis | Mix with donkey excrete and butter and paint | T |  |
|  | *Oryza sativa* L. | Ruz (A) | H | S | D | Diarrhea | Boil with water and drink the fluid | O | DTK155 |
|  | *Sorghum bicolor* (L.) Moench (DTK04) | Mashila (A) | H | S | F/D | Skin disease | Chew and spat | T | DTK04 |
| Polygonaceae | *Rumex nepalensis* Spreng. | Yewusha milas (A)  Terenchina (G) | H | L | F | Amoebiasis | Prepare the bud as a stew by adding all the ingredients and eat with bread | O | DTK42 |
|  |  |  |  | L | F | Intestinal parasites | Prepare the bud as a stew by adding all the ingredients and eat with bread | O |  |
|  |  |  |  | R | F | Malaria | Crush to remove the bitter part and then chew the remaining part | O |  |
|  |  |  |  | L | F | Malaria | Prepare the bud as a stew by adding all the ingredients and eat with bread | O |  |
| Polygonaceae | *Securidaca longepedunculata* Fresen. | Temenay (A)  Sekida (G) | T | R | F/D | Sudden sickness | Crush, put on fire, and inhale its smoke | I | DTK115 |
|  |  |  |  | R | D | Jaundice | Crush, mutilate the body 44 times by razer blood and paint the bleeding area as well as smell it | T and I |  |
|  |  |  |  | R | F/D | Febrile illness | Crush, put on fire, and inhale its smoke | I |  |
|  |  |  |  | R | F/D | Snake repellent | Put on a burning fire, the snake will not come around | - |  |
|  |  |  |  | R | F | Snakebite | Crush, homogenize with cold water, and drink or fire the tip of the root. Gently put it into the bitten area | O/T |  |
|  |  |  |  | R | D | Evil eye | Pound by mixing with the roots of *Tacazzea venosa*, *Withania somnifera, Sida rhombifolia*, and *Clerodendrum alatum* and put on fire and inhale | I |  |
| Rhamnaceae | *Rhamnus prinoides* L’Herit. | Gesho (A) | S | L | F | Evil spirit | Squeeze, collect with a cup and drink | O | DTK69 |
|  | *Ziziphus abyssinica* Hochst. ex A. Rich. | Ye-ahya gava (A)  Dequalgeba (Ag)  Hangugua (G) | S | R | F | Jaundice | Crush, mix with cold water and drink | O | DTK70 |
|  |  |  |  | R | F | Evil eye | Crush and tie | T |  |
|  | *Ziziphus spina-christi* (L.) Desf. | Gava (A)  Geba (Ag)  Siya (G) | S | SB | F | Uvulitis | Crush, mix with cold water and drink the fluid | O | DTK71 |
|  |  |  |  | R | F | Stomachache | Crush and drink the fluid | O |  |
|  |  |  |  | L | F | Ringworm | Paint the affected area with the unripe fruit | T |  |
|  |  |  |  | L | F | Dandruff | Pound, collect the fluid by sieve, add water, and paint | T |  |
|  |  |  |  | R | F | Malaria | Crush, add water, and drink the fluid | O |  |
|  |  |  |  | L | F | Spider poison | Squeeze and paint to the affected area | T |  |
| Rubiaceae | *Coffea arabica* L. | Bunna (A) | S | S | D | Wound healing | Roast, pound, mix with hair oil, paste on the affected area and tie | T | DTK126 |
|  | *Gardenia ternifolia* Schumach. & Thonn. | Gambilo (A)  Kota (G) | T | SB | F | Gum disease | Hold with teeth | O | DTK84 |
|  |  |  |  | F | F/D | Gum disease | Hold with teeth | O |  |
| Rutaceae | *Citrus × aurantiifolia*  (Christm.) Swingle | Lomi (A) | T | F | F/D | Nail fungus | Put on the finger | O | DTK75 |
|  | *Ruta chalepensis* L. | Tena Adam (A) | H | L | F | Coughing | Boil and drink the decoction as tea by adding sugar | O | DTK77 |
|  |  |  |  | L | F | Evil eye | Boil and drink the decoction as tea by adding sugar | O |  |
| Sapindaceae | *Pappea capensis* Eckl. & Zeyh. | Barkana (A) | T | R | F/D | Scorpion sting | Put the tip on fire and gently touch the area | T | DT151 |
|  |  |  |  | SB | F/D | Snakebite | Pound, mix with water, drink the filtrate, and then tie the residual | O/T |  |
|  |  |  |  | R | F/D | Snakebite | Put the tip on fire and gently touch the area | T |  |
|  |  |  |  | R | D | To eradicate devil | Put on fire and fumigate the house | - |  |
| Solanaceae | *Capsicum annuum* L. | Berberie (A) | H | R | D | Prevent miscarriage | Mix with water and eat | O | DTK137 |
|  |  |  |  | F | D | Spider poison | Mix with water and paint | T |  |
|  | *Capsicum frutescens*L. | Mitmita (A) | H | F | F | Diarrhea | Crush, add water and drink | O | DTK97 |
|  |  |  |  | R | F | Gastritis | Crush and eat with local bread called injera | O |  |
|  | *Datura stramonium* L. | Astenagir (A) | H | S | F/D | Toothache | Boil the part and inhale the smoke | I | DTK96 |
|  |  |  |  | L | F | Ringworm | Squeeze, and drop to the affected area | T |  |
|  |  |  |  | L | F | Tonsillitis | Squeeze, and drink | O |  |
|  |  |  |  | L | F | Scabies | Squeeze, mix with water and wash the body | T |  |
|  | *Solanum incanum* L. | Gever Embuay (A)  Ankakawa (G) | S | R | F | Snakebite | Chew and take the sap | O | DTK37 |
|  |  |  |  | F | F/D | Wound healing | Squeeze and paint | T |  |
|  |  |  |  | R | F | Stomachache | Chew and take the sap | O |  |
|  | *Solanum lycopersicum* L. | Timatim (A)  Bedenjala (G) | H | L | F | Bleeding wound | Squeeze and paste | T | DTK95 |
|  |  |  |  | L | F | Malaria | Squeeze, add water and drink | O |  |
|  | *Withania somnifera* (L.) Dunal | Gizewa (A) | S | L | F | Sudden sickness | Crush by mixing with *Zingiber officinale* and *Allium sativum*. Then smell or tie around the neck or mix with coffee and drink | I/T/O | DTK98 |
|  |  |  |  | L | F | Scabies | Squeeze and paste | T |  |
|  |  |  |  | R | F | Gum disease | Chew and take the fluid | O |  |
|  |  |  |  | L | F | Jaundice | Squeeze, mix with coffee powder, and drink the decoction | O |  |
|  |  |  |  | L | F | Stomachache | Squeeze add to coffee and drink | O |  |
|  |  |  |  | L | F | Common cold | Boil with the leaves of *Ocimum lamiifolium*. *Cordia africana* and drink the concotion | O |  |
|  |  |  |  | L | F | Covid | Boil with the leaves of *Ocimum lamiifolium*. *Cordia africana* and drink the concotion | O |  |
|  |  |  |  | L | F | Malaria | Boil with the leaves of *Zehneria scabra* and drink the concotion | O |  |
|  |  |  |  | L | F | Febrile illness | Boil and inhale the smoke or squeeze, mix with coffee powder, and drink the decoction | I/O |  |
| Vitaceae | *Capsicum annuum* L. | Berberie (A) | H | R | D | Prevent miscarriage | Mix with water and eat | O | DTK137 |
|  |  |  |  | F | D | Spider poison | Mix with water and paint | T |  |
|  | *Cissus quadrangularis* L. | Enzukaka (A) | H | R | F/D | Evil eye | Crush, mix with water and drink | O | DTK138 |
| [Zygophyllaceae](https://powo.science.kew.org/taxon/urn:lsid:ipni.org:names:30000513-2) | *Balanites aegyptiaca* (L.) Delile | Lalo (A)  Guaza (Ag)  Qota (G) | T | S | D | Intestinal parasite | Roast and eat | O | DTK88 |
|  |  |  |  | RB | F/D | Intestinal parasite | Crush, mix with water and drink | O |  |
|  |  |  |  | F | D | Intestinal parasite | Eat too much or mix it with water and drink | O |  |
|  |  |  |  | S | D | Diabetes mellitus | Roast and eat | O |  |

**Local name**: Amhara = A, Agew = Ag, Gumuz = G, **Habit = H**, Tree = T, Shrub = S, Climber = C, Herb = H, Liana = L, **Part used = PU**, Bulb = Bu, Flower = Fl, Fruit = F, Gum = G, Latex = La, Leaf = L, Resin = Re, Root = R, Root bark = RB, Seed = S, Seed pod = SP, Stem = St, Stem bark = SB, Tuber = Tw, Twig = T, Seed Oil = SO, **Condition of Part Used = CPU**, Fresh = F, Dry = D, Fresh/Dry = F/D, **Route of Administration = RA**, Auricular = A, Inhalation = I, Oral = O, Optical = Op, Topical = T, **Voucher numbers** = **Vouch. No.**
